# Playing With the Index of M-Theory

**DOI:** 10.1007/s00220-022-04479-7

**Published:** 2022-08-23

**Authors:** Michele Del Zotto, Nikita Nekrasov, Nicolò Piazzalunga, Maxim Zabzine

**Affiliations:** 1 Mathematics Institute, Uppsala University, Box 480, SE-75106 Uppsala, Sweden; 2 Department of Physics and Astronomy, Uppsala University, Box 516, SE-75120 Uppsala, Sweden; 3 Simons Center for Geometry and Physics, Stony Brook University, Stony Brook, NY 11794-3636 USA; 4 Center for Advanced Studies, Skoltech, Moscow, Russia; 5 Kharkevich Institute for Information Transmission Problems, Moscow, Russia

## Abstract

Motivated by M-theory, we study rank *n* K-theoretic Donaldson–Thomas theory on a toric threefold *X*. In the presence of compact four-cycles, we discuss how to include the contribution of D4-branes wrapping them. Combining this with a simple assumption on the (in)dependence on Coulomb moduli in the 7d theory, we show that the partition function factorizes and, when *X* is Calabi–Yau and it admits an ADE ruling, it reproduces the 5d master formula for the geometrically engineered theory on $A_{n-1}$ ALE space, thus extending the usual geometric engineering dictionary to $n>1$. We finally speculate about implications for instanton counting on Taub-NUT.

## Introduction

One can view the development of topological string theory as a journey from world sheet to target space: based on the realization [1] that the topological string free energy computes coefficients of effective action terms in the graviphoton background, the curve counting was re-interpreted [2–4] in terms of BPS state counting in string/M-theory, coming from M2-branes, with its genus-zero part giving a relativistic generalization of Seiberg–Witten theory [5]. Later on a tool was developed to compute the topological string partition function/instanton partition function in terms of box counting [6–9], which led to the connection with Donaldson–Thomas theory [10], geometric engineering [11], and spinning black holes [12].

Usual DT theory is obtained by placing a single D6-brane on a threefold *X* in type IIA string theory, which in M-theory becomes the Taub-NUT space. Similarly, for higher rank DT theory, we consider the *U*(*n*) theory on the worldvolume of *n* D6-branes wrapping $X\times S^1$. In the limit where we send the Taub-NUT radius to infinity, we obtain the $A_{n-1}$-type ALE space. At the same time a certain harmonic two-form that is $L^2$ on the Taub-NUT space becomes non-normalizable on the ALE space. Correspondingly the associated *U*(1) factor in the gauge group decouples. If $X$ is a canonical Calabi–Yau three-fold singularity, geometric engineering in M-theory assigns to it a five-dimensional superconformal field theory ${{{\mathcal {T}}}_{X}}$. Schematically,1.1Since $TN_n$ is non-compact, we can give boundary conditions at infinity to the scalar fields in ${{{\mathcal {T}}}_{X}}$. In particular, we can give vev to the operators parametrizing the Coulomb branch of ${{{\mathcal {T}}}_{X}}$. The latter correspond to the volumes of 2-cycles that arise from intersecting compact divisors in a smooth crepant resolution of $X$. If *X* is non compact, we also have compact 2-cycles that arise from intersecting compact divisors with non-compact ones: these correspond to mass deformations of ${{{\mathcal {T}}}_{X}}$, which are the only susy preserving relevant deformations in 5d. This is how the dependence on the Kähler parameters of *X* enters the 5d partition function of ${{{\mathcal {T}}}_{X}}$. We summarize our notations/dictionary, which will be explained later.1.2The two main achievements of this paper are as follows:

- given any toric threefold *X*, we extend usual Donaldson–Thomas theory in two directions: first by going to higher rank, namely from *U*(1) to *U*(*n*) gauge theory; second by including the contribution of D4-branes wrapping compact divisors. A simple assumption on the dependence on equivariant parameters allows us to prove a factorization property for this theory, which we call 7d master formula.
- if *X* is also Calabi–Yau and admits a geometric engineering limit, our 7d master formula matches the master formula for the geometrically engineered 5d gauge theory on $A_{n-1}$ space,^1^ which is the K-theoretic extension of usual 4d master formula.

Our motivation comes from M-theory (hence the title): although we will not be able to provide a full derivation of everything from M-theory, our construction has a clear 11d origin, which suggests the equality between two protected quantities as they come from different reductions of the same 11d object. Conversely, our computations can be regarded as an equivariant test of M-theory. Nevertheless, the main statements and conjectures of our paper can be formulated in a mathematically rigorous way, ignoring their physical origin.

Our story is in many ways an extension of the work [13], where higher rank DT theory was presented, and its connection to the index of M-theory on Calabi–Yau fivefolds was discussed. We explore the effect of additional topological sectors, allowing for sheaves with nontrivial $c_1$ on the threefold side, and the fluxes through the 2-cycles on the twofold side. Certain bits of our story appeared previously in the work [14], where the relation between the instantons on ALE and ALF spaces was studied, and hints at a DT-like interpretation were pointed out. Physically, our approach includes in a crucial way the effects of the *D*4-branes, which were not considered in the abovementioned papers.

### Plan

In Sect. 2 we review the M-theory background that underlies our computations. Although some aspects of the story are well-known, the full lift of the equivariant $\Omega$-background, including the $G_4$ flux, that would allow to perform the localization calculations directly in M-theory, is not. Some of our considerations therefore remain qualitative.^2^

In Sect. 3 we review the instanton counting in 4+1d on non-compact toric manifolds, in particular we present a straightforward extension to 4+1d of the 4d master formula. We discuss the simplest cases, namely the vanishing Chern–Simons level and no matter, but we believe our findings are valid more generally. We also compare the ALE and ALF cases, and present a toy model computation in detail.

In Sect. 4 we review the Donaldson–Thomas theory on a toric threefold *X*, and extend it to the higher rank. We recall useful facts from toric geometry and the DT/PT correspondence for local *X*.

In Sect. 5 we combine the previous ingredients with the Coulomb-independence hypothesis, and explain how to introduce the *D*4-branes. The main result there is the 7d master formula. This can be seen either mathematically as a factorization property for a generic toric threefold *X*, or as an extension of the usual geometric engineering if *X* is Calabi–Yau and engineers a gauge theory. In the latter case, the 7d master formula matches exactly the 5d one for the corresponding theory.

While in Sect. 5 we keep the discussion general, in Sect. 6 we try to give as many details as possible for a few relevant examples. After spelling out some details of the geometric engineering dictionary, we test our findings on some of the geometries engineering the *SU*(*N*) gauge theory with zero CS level for $N=2,3$.

## M-Theory Setup

We review the M-theory framework that motivates our paper [13, 16]. We begin with an overview of the general structure, and then discuss the special class of backgrounds that give rise to the examples we consider in this paper.

### An identity from Calabi–Yau fivefolds

M-theory admits supersymmetric compactifications on Calabi–Yau 5-folds (CY5) of the form

2.1 $$\begin{aligned} M_{11} = {{\mathbb {R}}}\times M_{10} \end{aligned}$$

which for generic CY5 preserve two supercharges [17].

In our paper we consider manifolds $M_{10}$ admitting isometries. In this context we can define the twisted Witten index

2.2 $$\begin{aligned} \mathrm{Tr} (-1)^{F} g = Z (S^{1} {{\tilde{\times }}} M_{10}) \end{aligned}$$

where $S^{1} {{\tilde{\times }}} M_{10}$ denotes a fiber bundle over $S^1$ with fiber $M_{10}$, which is the cylinder of the isometry map $g: M_{10} \rightarrow M_{10}$. We assume *g* to commute with some supercharge.

Of course, for compact $M_{10}$, this makes no sense, since, firstly, one is supposed to integrate over all metrics on $M_{10}$, and secondly, all diffeomorphisms of $M_{10}$, including the rare instances of isometries of a fluctuating metric, are gauge symmetries, and, therefore, act trivially on the physical states. Hence, we assume $M_{10}$ to be a non-compact space, asymptotically approaching a fixed CY5 with nontrivial isometries. These isometries are then treated as global symmetries.

We denote by ${\mathcal {T}}_{M_{d}}$ the $(11 - d)$-dimensional theory obtained from M-theory on ${{\mathbb {R}}}^{1,10-d} \times M_d$. If $M_d$ is non-compact ${\mathcal {T}}_{M_{d}}$ is non-gravitational. More precisely, the gravitational physics is fully eleven-dimensional, while the dynamics of the $11-d$-dimensional (localized) degrees of freedom takes place in the fixed gravitational background. Actually, as explained in [18] certain gauge-like degrees of freedom can be interpreted as topology changes, thus representing the gravitational dynamics using supersymmetric gauge theory (this could be compared to the AdS/CFT duality, in a topological context).

When $m+k=5$, the index Eq. (2.2) can be interpreted in two ways: on the one hand we have the partition function of ${\mathcal {T}}_{M_{2k}}$ on $S^1 \times M_{2m}$, on the other we have the partition function of ${\mathcal {T}}_{M_{2m}}$ on $S^1 \times M_{2k}$. These have to agree, giving the identity

2.3 $$\begin{aligned} Z^{(11-2k)d}_{{\mathcal {T}}_{M_{2k}}} (S^1 \times M_{2m}) =Z ({S^1 \times M_{2k} \times M_{2m}}) = Z^{(11-2m)d}_{{\mathcal {T}}_{M_{2m}}}(S^1 \times M_{2k}) \end{aligned}$$

### A 7d/5d correspondence

The CY5 of our interest are a product

2.4 $$\begin{aligned} M_{10} = M_4 \times M_6 \end{aligned}$$

where $M_4$ is either the charge *n* Taub-NUT space or an ALE space and $M_6=X$ is a CY3 singularity.^3^ The $M_4$ spaces at their most singular point in the Kähler moduli engineer 7d maximally supersymmetric Yang–Mills theories in M-theory [19]. The space $X$ engineers a 5d SCFT ${{{\mathcal {T}}}_{X}}$ in M-theory [20, 21]. The resulting geometries preserve 4 supercharges and both give rise to non-gravitational theories. We are led to an equation of the form

2.5 $$\begin{aligned} Z^{7d}_{{\mathcal {T}}_{M_4}}(S^1_\beta \times M_6) = Z^{5d}_{{\mathcal {T}}_{M_6}}(S^1_\beta \times M_4) \end{aligned}$$

where the partition functions are interpreted as twisted Witten indices. Since both spaces are non-compact, these partition functions depend on choices of boundary conditions at infinity.

### A heuristic argument: topological bootstrap

In the case $M_4 = TN_n$ we have a relation with higher rank DT theory, building upon the classical duality among M-theory on $S^1_\beta \times TN_n\times X$ and IIA on $S^1_\beta \times {{\mathbb {R}}}^3 \times X$ with *n* D6 branes wrapping $S^1_\beta \times X$, and exploiting the Taub-NUT circle as the M-theory circle.

One could add D4 branes wrapping $S^1_\beta \times D$, where *D* is a holomorphic 4-cycle of *X*,

2.6 $$\begin{aligned} {\text {vol}} D = \int _{D} \omega \wedge \omega , \end{aligned}$$

where $\omega$ is the Kahler form of *X*. These are non-supersymmetric at first sight: indeed for $X = {{\mathbb {C}}}^3$ one such state would correspond to a parallel system of D4–D6 branes, which breaks supersymmetry as the number of Dirichlet–Neumann directions is not a multiple of 4. However, in that context the D4-brane dissolves into flux for the D6-brane. Therefore we could in principle include these configurations at the price of dissolving the D4-branes into localized flux in our background. Dualizing these D4-branes back to M-theory we obtain M5-branes wrapping the Taub-NUT circle, which is fibered and shrinks at the position of the D6-branes. These M5-branes are localized where the Taub-NUT circle shrinks and dissolve in $G_4$ flux localized in the complement of such region. Depending on how we do the reduction, we have two possible ansatzes

2.7 $$\begin{aligned} \begin{aligned} G_4&\sim F^{7d}_a \wedge B^a + m_{i,a} PD_X[D^i] \wedge B^a \\ G_4&\sim F^{5d}_i \wedge PD_X[D^i] + m_{i,a} PD_X[D^i] \wedge B^a \end{aligned} \end{aligned}$$

where $m_{i,a}$ is the number of M5 branes that are wrapped on $C_a$ (see Appendix B for notations) inside $TN_n$ and the other fields represent the KK modes corresponding to the field strengths of the 7d and 5d theories, respectively. Here $PD_X[D^i]$ stands for Poincaré dual of compact four cycles $D^i$ in *X*. This suggests that M5-branes wrapping the Taub-NUT circle and a compact divisor within the CY3 can be interpreted as nontrivial first Chern classes for either of the curvatures of the field theories in the 5d/7d correspondence. Indeed, we have

2.8 $$\begin{aligned} \begin{aligned} G_4&\sim (F^{7d}_a + m_{i,a} PD_X[D^i]) \wedge B^a \\ G_4&\sim (F^{5d}_i + m_{i,a} B^a) \wedge PD_X[D^i] \end{aligned} \end{aligned}$$

and each non zero $m_{i,a}$ can be absorbed as a non-trivial first Chern class for the curvatures on the 7d and the 5d sides. This discussion is purely heuristic and at the moment we do not have enough tools to derive 5d/7d actions from 11d M-theory perspective. However we know that the properly defined volume ${{\mathcal {F}}}(t)$ of CY (triple intersection number of *X*) can be interpreted as the prepotential of the rigid supersymmetric five-dimensional theory [20–22].

The bootstrap approach to quantum field theory of [23] recently has led to great advances in the quantitative analysis of conformal field theories in three and four dimensions (see e.g., [24, 25]). The conformal bootstrap in two dimensions, at the level of a 4-point correlation function

2.9 $$\begin{aligned} \langle {{\mathcal {O}}}_{1}(x_1) {{\mathcal {O}}}_{2}(x_2) {{\mathcal {O}}}_{3}(x_3) {{\mathcal {O}}}_{4}(x_4) \rangle \end{aligned}$$

is the requirement of the equality of two expansions, one in the limit $x_{2} \rightarrow x_{1}$ (which is equivalent, thanks to conformal invariance, to $x_{3} \rightarrow x_{4}$ limit), and another in the limit, e.g., $x_{3} \rightarrow x_{2}$ (equivalent to $x_{4} \rightarrow x_{1}$). These expansions correspond to the respective *s*- and *t*-channel tree diagrams (labelling the 4-point conformal blocks). In the context of toric geometry, similar tree diagrams describe the two phases of the resolved conifold $X = \left[ {{\mathcal {O}}}(-1) \oplus {{\mathcal {O}}}(-1) \longrightarrow {{\mathbb {C}}{\mathbb {P}}}^{1} \right]$, which can be described as the symplectic quotient of ${{{\mathbb {C}}}}^{4}$ by *U*(1):

2.10 $$\begin{aligned} |z_{1}|^2 + |z_{2}|^2 - |z_{3}|^2 - |z_{4}|^2 = r, \quad \left( z_{1}, z_{2}, z_{3}, z_{4} \right) \sim \left( e^{\mathrm i\theta } z_{1}, e^{\mathrm i\theta } z_{2}, e^{-\mathrm i\theta }z_{3}, e^{-\mathrm i\theta }z_{4} \right) . \end{aligned}$$

For $r \ne 0$, the edges of the toric polytope $\Delta _{X}$ (not to be confused with the 1-skeleton ${\Delta }_{X}^{(1)}$ used in this paper) consist of four semi-infinite axes $l_{1}, l_{2}, l_{3}, l_{4}$ and one finite interval *c*. For $r > 0$ these are $l_{1} = \{ z_{3} = z_{1} = 0\} \cup l_{2} = \{ z_{4} = z_{1} = 0 \} \amalg l_{3} = \{ z_{3} = z_{2} = 0 \} \cup l_{4} = \{ z_{4} = z_{2} = 0 \}$, and $c = \{ z_{3} = z_{4} = 0\}$, respectively. For $r< 0$ the geometry is identical with $(z_{1}, z_{2}) \leftrightarrow (z_{3}, z_{4})$. The generating function of Gromov–Witten invariants admits the analytic continuation $r \rightarrow - r$, so that the essential part of the instanton counting agrees for *s*- and *t*-channels. Perhaps closer in spirit to the bootstrap of CFT is the associativity WDVV equation obeyed by the genus-zero Gromov–Witten invariants [26].

We call the conjectured equality of the 5*d*/7*d* perspectives the *topological bootstrap*. We imagine it also corresponds to some homotopy between the “large TN - small CY” and the “small TN - large CY” geometries, akin to the flop transition $r \ll 0 \rightarrow r\gg 0$ of the resolved conifold. The validity of our conjecture strengthens the belief in the existence of the underlying 11*d* theory.

## 5D Theory on $TN_n \times S^1_\beta$

We review and discuss the properties of 4d and 5d instanton partition functions on non-compact manifolds with $T^2$-action. In particular we are interested in non-compact toric ALE spaces of type $A_{n-1}$ and their cousins $TN_n$, the multi-Taub-NUT spaces.

Let us start with the basic setup. In 4d a ${{\mathcal {N}}}=2$ gauge theory can be twisted and placed on arbitrary manifolds. After twisting, the theory can be recast as a cohomological field theory, which is known as Donaldson–Witten theory. If the underlying manifold admits a $T^2$ action, then one can define equivariant Donaldson–Witten theory. Originally equivariant Donaldson–Witten theory has been discussed on ${{{\mathbb {C}}}}^2$ [27–30] and this effort has resulted in the definition of the instanton partition function [6, 7]. For pure *U*(*N*) ${{\mathcal {N}}}=2$ gauge theory on ${{{\mathbb {C}}}}^2$, the full partition function is given by

3.1 $$\begin{aligned} Z^{4d}_{U(N)} ({{{\mathbb {C}}}}^2; z, \vec {\varphi }, \epsilon _4, \epsilon _5) = Z^{4d}_{\mathrm{cl}} Z^{4d}_{\mathrm{1-loop}} \sum _{l=0}^{\infty } z^l ~\mathrm{vol}_l (\vec {\varphi }, \epsilon _4, \epsilon _5), \end{aligned}$$

where ${\text {vol}}_l (\vec {\varphi }, \epsilon _4, \epsilon _5)$ is the equivariant volume of the moduli space of instantons of charge *l* and $Z^{4d}_{\mathrm{cl}}$, $Z^{4d}_{\mathrm{1-loop}}$ stand for the classical and 1-loop parts correspondingly. Here the parameters $(\vec {\varphi }, \epsilon _4, \epsilon _5)$ are the equivariant parameters for the $T^{N+2}$ action on the moduli space of instantons, where $\vec {\varphi }$ stands for the constant gauge transformations (one refers to them as Coulomb branch parameters) and $(\epsilon _4, \epsilon _5)$ stand for $T^2$-rotations of ${{{\mathbb {C}}}}^2$. The parameter *z* is an instanton counting parameter. The 4d ${{\mathcal {N}}}=2$ gauge theory on ${{{\mathbb {C}}}}^2$ has a natural 5d lift to ${{{\mathbb {C}}}}^2 \times S^1_{\beta }$ and the partition function corresponds to the index

3.2 $$\begin{aligned} Z^{5d}_{U(N)} ({{{\mathbb {C}}}}^2 \times S^1_\beta ; z, \vec {b}, q_4, q_5) = Z^{5d}_{\mathrm{cl}} Z^{5d}_{\mathrm{1-loop}} \sum _{l=0}^{\infty } z^l ~\mathrm{ind}_l (\vec {b}, q_4, q_5), \end{aligned}$$

where $\mathrm{ind}_l (\vec {b}, q_4, q_5)$ stands for the equivariant index of the Dirac operator on the moduli space of instantons of charge *l* and $\vec {b} = e^{\beta \vec {\varphi }}$, $q_4 =e^{\beta \epsilon _4}$, $q_5= e^{\beta \epsilon _5}$. The index $\mathrm{ind}_l$ can be written as an integral of the equivariant A-roof genus over the moduli space of instantons. In 5d one can add a Chern–Simons term. The partition function on ${{\mathbb {C}}}^2$ and ${{\mathbb {C}}}^2 \times S^1_\beta$ has been generalized to a wide class of ${\mathcal {N}}=2$ supersymmetric theories and it has been studied extensively in different contexts, see Ref. [31] for a review.

The equivariant Donaldson–Witten theory can be defined on any four manifold $M_4$ that admits isometries and the most interesting case is when $M_4$ admits a $T^2$ action. There are two distinct cases of such theories: the case of non-compact and compact $M_4$. Here we concentrate on the case of non-compact four manifold with $T^2$-action. The 4d and 5d partition functions can be defined in the same way as in Eqs. (3.1) and (3.2) if we know the explicit construction of the corresponding instanton moduli space. On general grounds we expect the appropriate torus action on the instanton moduli space (e.g., $T^{N+2}$ action for the *U*(*N*) theory). The main new feature is that the partition function may depend on more parameters associated to extra labels related to the moduli spaces and the underlying geometry of $M_4$. In the partition function different configurations are weighted by the classical term

3.3 $$\begin{aligned} \int _{M_4} e^{H + \omega }~ \mathrm{ch} (F), \end{aligned}$$

which in the path integral gets extended to the appropriate equivariant observable (in 5d on $M_4 \times S^1_\beta$ we can also add Chern–Simons terms). Here $\omega$ is an invariant symplectic form on $M_4$ and *H* the corresponding Hamiltonian for the $T^2$-action. In principle, one can construct more general observables but this is not relevant for our discussion.

If $M_4$ is a toric variety then it can be glued from ${{\mathbb {C}}}^2$ pieces. The corresponding 4d master formula for non-compact toric varieties [32–34] takes the form

3.4 $$\begin{aligned} \boxed { Z^{4d}_{SU(N)} (M_4; z, \vec {\varphi }, \epsilon _4, \epsilon _5) = \mathop \sum \limits _{(\vec {h}_1, \ldots , \vec {h}_p) \in {{\mathbb {Z}}}^{(N-1)p}} \prod _{i=1}^k Z^{4d}_{SU(N)} \Big ({{{\mathbb {C}}}}^2; z, \vec {\varphi } + \sum _{j=1}^k \phi ^{(i)}_j \vec {h}_j, \epsilon ^{(i)}_4, \epsilon ^{(i)}_5 \Big ) } \nonumber \\ \end{aligned}$$

where we are interested in *SU*(*N*) gauge theory. Here we deal with a smooth toric variety with *k* fixed points under the $T^2$ action and for every fixed point there exists a $T^2$-invariant open affine neighborhood isomorphic to ${{\mathbb {C}}}^2$, with $\epsilon ^{(i)}_4$, $\epsilon ^{(i)}_5$ encoding the $T^2$-action at fixed point *i*. The integers $\vec {h}_j$ ($j=1, \ldots , p= \dim H^2_c(M_4, {{\mathbb {Z}}})$) correspond to the so-called fluxes, which are labeled by compactly supported $H^2_c (M_4, {{\mathbb {Z}}})$ in every Cartan direction. In Eq. (3.4), the weights $\phi ^{(i)}_j$ are constructed from toric data. Equation (3.4) admits different refinements, for example we can fix the holonomy at infinity, in case a boundary of the toric space has non-trivial topology (allowing different flat connections at infinity). We aren’t interested in such refinements and leave them aside. Our main interest are *SU*(*N*) gauge theories, so we assume the traceless condition for $\vec {\varphi }$ and for every $\vec {h}_j$ with the appropriate invariant scalar product.

We follow the review [35], where one may find further mathematical details. We assume that Eq. (3.4) has a straightforward 5d lift. In 5d Chern–Simons terms can be introduced, but we mainly ignore them to avoid cluttering in our formulas.

We are interested in two types of spaces: ALE spaces of type $A_{n-1}$ and multi-Taub-NUT spaces $TN_n$, which are both hyperKähler and admit $T^2$ isometries (provided that the centres of these spaces are aligned). Although $A_{n-1}$ is a limit of $TN_n$, their instanton partition functions may differ, since asymptotically they look different. Let us start from the spaces $A_{n-1}$, which are examples of non-compact toric varieties.

### ALE spaces of $A_{n-1}$ type

ALE spaces of type $A_{n-1}$ are hyperKähler four-manifolds that can be thought of as deformation (resolution) of the quotient ${{\mathbb {C}}}^2/{{\mathbb {Z}}}_n$, with ${{\mathbb {Z}}}_n$ being understood as subgroup of *SU*(2) acting isometrically on ${{\mathbb {C}}}^2$. We collect some basic properties of $A_{n-1}$ spaces in Appendix A. In what follows we assume that the metric on $A_{n-1}$ has a $T^2$ isometry and thus the centres are aligned.

There are two approaches to instanton partition functions on $A_{n-1}$. In the first approach one constructs the instanton moduli space directly, and this was done by Kronheimer and Nakajima [36] by considering ADHM data invariant under ${{\mathbb {Z}}}_{n}$. Later Nakajima [37] described them in terms of Nakajima quiver varieties. Thus one can define the instanton partition function on the $A_{n-1}$ space as the partition function for an appropriate quiver variety. The second approach is based on the fact that the resolved $A_{n-1}$ space is a toric variety and thus the full partition function on $A_{n-1}$ can be glued from ${{\mathbb {C}}}^2$ pieces. Physically the two approaches should produce the same result as long as the partition function is independent from the sizes of resolved cycles. However, this relation has not been proved, as far as we know.

Here we follow the second approach and assume that Eq. (3.4) gives the full result for the $A_{n-1}$ space. Our goal is to write the 5d version of this formula with all toric data spelled out for $A_{n-1}$ (for a review see Appendix A). Gluing $A_{n-1}$ from ${{\mathbb {C}}}^2$ pieces, the full 5d partition function takes the form

3.5 $$\begin{aligned}&Z_{SU(N)}^{5d} (A_{n-1} \times S^1; z, \vec {b}, q_4, q_5) \nonumber \\&\quad = \sum _{(\vec {h}_1, \ldots , \vec {h}_{n-1}) \in ({{\mathbb {Z}}}^{(N-1)})^{n-1}} \prod _{i=1}^n Z^{5d}_{SU(N)} ({{{\mathbb {C}}}}^2 \times S^1; z, \vec {b}^{(i)}, q^{(i)}_4, q^{(i)}_5) \end{aligned}$$

where we are ignoring the Chern–Simons level. Here $q_4= e^{\beta \epsilon _4}$, $q_5= e^{\beta \epsilon _5}$ are global parameters associated to the $T^2$ action, while the local toric parameters $q_{4}^{(i)}= e^{\beta \epsilon _4^{(i)}}$, $q_5^{(i)}= e^{\beta \epsilon _5^{(i)}}$ for fixed point *i* are defined as

3.6 $$\begin{aligned} q_{4}^{(i)} = q_4^{n-i+1} q_5^{1-i},\quad q_5^{(i)} = q_4^{i-n} q_5^{i}, \end{aligned}$$

and these expressions can be read off from the toric data, see Eq. (A.22). From global $\vec {b} = ({b_\alpha })$ with $\alpha = 1, \ldots , N$ being Cartan direction and $b_\alpha = e^{\beta \varphi _\alpha }$, the local data are defined as

3.7 $$\begin{aligned} b_\alpha ^{(i)} = b_\alpha (q_4^{(i)})^{h_{i,\alpha }} (q_5^{(i)})^{h_{(i-1),\alpha }} = b_\alpha (q_4^{n-i} q_5^{-i})^{h_{i,\alpha } -h_{(i-1),\alpha }} (q_4 q_5)^{h_{i,\alpha }}, \end{aligned}$$

where $\vec {h}_i = \{h_{i, \alpha }\}$ are integers parametrized by Cartan direction $\alpha$ and fixed point *i*. Within geometric engineering, we are interested in *SU*(*N*) theories, thus in the above formulas we impose the trace condition both for the Cartan parameters and for the fluxes, $h_{0,\alpha } =0 =h_{n,\alpha }$.

For the sake of our forthcoming discussion, classical terms for $A_{n-1}$ geometry are glued as

3.8 $$\begin{aligned} \beta ^{-1} \log \Big (Z^{5d}_{\mathrm{cl}} (A_{n-1}\times S^1)\Big )= & {} \sum _{i=1}^n \frac{\Big \langle \vec \varphi + \vec {h}_i \epsilon _4^{(i)} + \vec {h}_{i-1} \epsilon _5^{(i)}, \vec \varphi + \vec {h}_i \epsilon _4^{(i)} + \vec {h}_{i-1} \epsilon _5^{(i)} \Big \rangle }{ \epsilon _4^{(i)} \epsilon _5^{(i)} } \nonumber \\= & {} \frac{\langle \vec \varphi , \vec \varphi \rangle }{n \epsilon _4 \epsilon _5} + \sum _{i=1}^n \Big ( 2 \langle \vec {h}_{i}, \vec {h}_{i-1}\rangle - 2 \langle \vec {h}_{i}, \vec {h}_i \rangle \Big ) \nonumber \\= & {} \frac{\langle \vec \varphi , \vec \varphi \rangle }{n \epsilon _4 \epsilon _5} +{{\mathcal {C}}}_{ij} \langle \vec {h}_i ,\vec {h}_j\rangle \end{aligned}$$

where $\langle ,\rangle$ stands for the Lie algebra pairing and ${{\mathcal {C}}}_{ij}$ is defined in Eq. (B.9) (it is related to the geometry of $A_{n-1}$).

### Multi Taub-NUT spaces $TN_n$

The cousins of ALE spaces of $A_{n-1}$ type are ALF spaces, the multi center Taub-NUT spaces $TN_n$. They are four-dimensional hyperKähler spaces asymptotic at infinity to ${{\mathbb {R}}}^3 \times S^1$, with *R* the radius of this circle. Close to the origin $TN_n$ looks like the $A_{n-1}$ space. Thus $TN_n$ can be thought of as hyperKähler deformation of $A_{n-1}$ with deformation parameter $R^{-1}$. Taking *R* to infinity reduces the $TN_n$ hyperKähler metric to the $A_{n-1}$ hyperKähler metric.

As far as we are aware there is no formula for the instanton partition function on $TN_n$. In 2008 Cherkis [38] initiated a systematic study of the instanton moduli spaces for *U*(*N*) gauge theory on $TN_n$. The instanton moduli space on $TN_n$ is labeled by the following charges [39]: the second Chern class $c_2$, a collection of *n* first Chern classes^4^$c_1$ and a collection of *N* non-negative integer monopole charges $(j_1, \ldots , j_N)$. The main novelty is the appearance of monopole charges related to the fact that the self-duality condition is reduced to the monopole equation at infinity. The bow diagrams (a generalization of quiver diagrams) encode an ADHM-like construction for the moduli space of instantons [39]. We are unaware of any direct equivariant calculation for this construction. However, if we restrict to the zero-monopole sector then the moduli space of instantons on $TN_n$ and on $A_{n-1}$ are related. They are not isomorphic as hyperKähler manifolds but they are isomorphic as complex symplectic varieties [38, 40]. Our guess is that, since the partition function is not sensitive to the spacetime metric as long as the isometries are preserved, the equivariant volume is the same for both spaces and thus the instanton partition function for $TN_n$ in the zero monopole sector coincides with the partition function for $A_{n-1}$. In the next subsection we offer a toy calculation that may indicate this is true. Again, the two spaces $TN_n$ and $A_{n-1}$ are different as hyperKähler spaces, but isomorphic as complex varieties, the isomorphism being $T^2$-equivariant. We calculate the $T^2$-equivariant volume for both $TN_n$ and $A_{n-1}$ and show that they coincide. This is an indication that a similar result is true for the moduli spaces of $TN_n$ (zero monopole sector) and $A_{n-1}$.

### Toy calculation

We evaluate the equivariant volume of $TN_n$ with respect to the $T^2$ action and show that it agrees with that of $A_{n-1}$. The original idea appeared in the work [28], where part of the calculation was presented. Here we spell out the details and use the full $T^2$ action on $TN_n$ with one *U*(1) being the triholomorphic action and another *U*(1) the non-triholomorphic action (for the metric to have these symmetries we require the centres to be aligned).

We follow Ref. [41] in the explicit construction of $TN_n$ as a hyperKähler quotient. With the standard quaternionic notations $i^2 = j^2 = k^2 = ijk = -1$, let ${\mathcal {M}} = {\mathbb {H}}^n \times {\mathbb {H}}$, with coordinates $q_a$ and *w*, for $a=1,\ldots , n$, with $G={{\mathbb {R}}}^n$ action

3.9 $$\begin{aligned} q_a \rightarrow q_a e^{i t_a}, \quad w \rightarrow w + R \sum _a t_a \end{aligned}$$

with $R \in {{\mathbb {R}}}$. Take hyperKähler quotient $TN_n = \mu ^{-1}(\varvec{\zeta })/G$ with moment maps

3.10 $$\begin{aligned} \mu _a = \frac{1}{2} {\varvec{r}}_a + R {\varvec{y}}, \end{aligned}$$

where $q_a = a_a e^{i \psi _a/2}$, ${\varvec{r}}_a = q_a i {\bar{q}}_a$ and $w = y + {\varvec{y}}$. Here *y* is real and $a_a,{\varvec{y}}$ pure quaternions. Let ${\varvec{y}} = \frac{{\varvec{r}}}{2R}$, $\varvec{\zeta }_a = \frac{1}{2} {\varvec{x}}_a$ and define

3.11 $$\begin{aligned} \chi _a = \chi ({\varvec{r}}_a) = \frac{da_a ia_a - a_ai da_a}{|a_a|^2}, \end{aligned}$$

so that $\chi = \sum _a \chi _a$ satisfies $d\chi = \star _3 dV$ with flat 3d metric and

3.12 $$\begin{aligned} V = \frac{1}{R^2} + \sum _{a=1}^n \frac{1}{|{\varvec{x}}_a - {\varvec{r}}|}. \end{aligned}$$

With $\tau = \sum _a \psi _a - \frac{2}{R} y$, the metric

3.13 $$\begin{aligned} ds^2 = \sum _a dq_a \otimes d{\bar{q}}_a + dw \otimes d{\bar{w}} \end{aligned}$$

becomes (after imposing moment map equations)

3.14 $$\begin{aligned} ds^2 = \frac{1}{4} V d{\varvec{r}} \otimes d \overline{{\varvec{r}}} + \frac{1}{4} \sum _{a=1}^n |{\varvec{x}}_a - {\varvec{r}}| (d\psi _a + \chi _a)^2 + dy^2. \end{aligned}$$

The vector fields generating the *G*-action are

3.15 $$\begin{aligned} v_a = 2 \frac{\partial }{\partial \psi _a} + R \partial _y \end{aligned}$$

and requiring the metric to satisfy $g(v_a,X)=0$ for any *a* and *X* yields

3.16 $$\begin{aligned} |{\varvec{x}}_a - {\varvec{r}}| (d\psi _a + \chi _a) + 2 R dy = 0. \end{aligned}$$

Plugging this back, finally

3.17 $$\begin{aligned} ds^2 = \frac{1}{4} V d{\varvec{r}} \otimes d \overline{{\varvec{r}}} + \frac{1}{4} V^{-1} (d\tau +\chi )^2 \end{aligned}$$

With $r_a = |{\varvec{r}}_a|$, we have

3.18 $$\begin{aligned} dq_a \wedge d {\bar{q}}_a = \frac{1}{4r_a} (r_a\chi _a-d{\varvec{r}}_a) \wedge (r_a\chi _a+d{\varvec{r}}_a) +\frac{1}{2} d\psi _a \wedge d{\varvec{r}}_a \end{aligned}$$

so that Kahler forms

3.19 $$\begin{aligned} i \omega _I + j \omega _J + k \omega _K = -\frac{1}{2} \sum _{a=1}^n dq_a \wedge d {\bar{q}}_a -\frac{1}{2} dw \wedge d {\bar{w}} \end{aligned}$$

become (using moment maps)

3.20 $$\begin{aligned} \sum _{a=1}^n dq_a \wedge d {\bar{q}}_a + dw \wedge d {\bar{w}} = -\frac{1}{4} V d{\varvec{r}} \wedge d{\varvec{r}} -\frac{1}{2} (d\tau +\chi ) \wedge d{\varvec{r}} \end{aligned}$$

In complex coordinates $q_a = z_a + w_a j$, ${\varvec{y}} = x_r i + x_c k$ we have

3.21 $$\begin{aligned} i \omega _I = i dy \wedge dx_r -\frac{1}{2} dx_c \wedge d{\bar{x}}_c -\frac{1}{2} \sum _a dz_a \wedge d{\bar{z}}_a + dw_a \wedge d{\bar{w}}_a \end{aligned}$$

while moment maps become

3.22 $$\begin{aligned} \mu _a = i\left( \frac{1}{2} (|z_a|^2-|w_a|^2) + R x_r \right) + (R x_c-z_a w_a) k \end{aligned}$$

The triholomorphic $U(1)_t$ acts as $\tau \rightarrow \tau + 2n \alpha$ with moment map $\mu _t = \frac{n}{2} {\varvec{r}}$. If $\varvec{\zeta }_a= i \zeta _a$ with $\zeta _a \in {{\mathbb {R}}}$, so that centers are aligned, there’s a non-triholomorphic $U(1)_n$ acting as $q_a \rightarrow e^{i \alpha } q_a$, $w \rightarrow e^{i \alpha } w e^{-i \alpha }$, which implies $z_a \rightarrow e^{i \alpha } z_a$, $w_a \rightarrow e^{i \alpha } w_a$, $x_r \rightarrow x_r$, $x_c \rightarrow e^{2i\alpha } x_c$, with Hamiltonian

3.23 $$\begin{aligned} H_n = |x_c|^2 + \frac{1}{2} \sum _a |z_a|^2+|w_a|^2 \end{aligned}$$

Up to a constant, the part of $\mu _t$ preserved by $U(1)_n$ is

3.24 $$\begin{aligned} H_t = \sum _a \left( R x_r - \zeta _a \right) \end{aligned}$$

and the equivariant volume is

3.25 $$\begin{aligned} {\text {vol}} (TN_{n}) := \int _{TN_n} dvol_g \exp (-\epsilon _n H_n -\epsilon _t H_t) \end{aligned}$$

We have (using moment maps)

3.26 $$\begin{aligned} r_a = |z_a|^2 + |w_a|^2 = 2 \sqrt{ (\zeta _a-R x_r)^2+| R x_c|^2 } \end{aligned}$$

With $R x_c = \rho e^{i \theta }$, we have

3.27 $$\begin{aligned} \frac{\partial H_n}{\partial \rho } = 2 \rho V \end{aligned}$$

and if we require $\Re \epsilon _n >0$ we see that the volume is independent of *R* and it becomes

3.28 $$\begin{aligned} {\text {vol}} (TN_{n})= \frac{2 \pi ^2}{\epsilon _n} \int _{-\infty }^{+\infty } d\sigma \exp (-\epsilon _n \sum _a |\sigma -\zeta _a| -\epsilon _t \sum _a (\sigma -\zeta _a)) \end{aligned}$$

Let’s take $\Re \epsilon _n > | \Re \epsilon _t|$ and use analytic continuation. By ordering $\zeta _1< \zeta _2< \ldots < \zeta _n$ we get

3.29 $$\begin{aligned} {\text {vol}} (TN_{n}) / (4\pi ^2)= & {} \frac{1}{n(\epsilon _n - \epsilon _t)(\epsilon _n + \epsilon _t)} - \frac{1}{2} \sum _{i=1}^n (\zeta _i-\frac{\zeta _*}{n} )^2 - \frac{\epsilon _n}{3!} \sum _{i<j} (\zeta _i-\zeta _j)^3\nonumber \\&+ \frac{n\epsilon _t}{3!} \sum _{i=1}^n (\zeta _i-\frac{\zeta _*}{n} )^3 + O(\epsilon ^2), \end{aligned}$$

where $\zeta _*= \sum _i \zeta _i$. This agrees with Eq. (A.27), if we set $\epsilon _t = \frac{1}{2} (\epsilon _5 - \epsilon _4)$, $\epsilon _n = \frac{1}{2} (\epsilon _4 + \epsilon _5)$ and $\zeta _a - \frac{\zeta _*}{n} = - \alpha _a$. The first two terms agree with Ref. [28]. The volume of $TN_n$ can be an inspiration for the definition of 7d classical action Eq. (4.36).

## DT Theory on CY

In this section, we review [42, 43] Donaldson–Thomas theory, focusing on toric Calabi–Yau^5^ threefolds *X*, and extend it to higher rank *n*. From a practical perspective, we view both equivariant DT theory in 3 complex dimensions and equivariant Donaldson–Witten theory in 2 complex dimensions as box counting problems [13].

### The setup

Our type IIA setup consists of *n* D6-branes (treated as background) wrapping $X \times S^1$, with lower-dimensional branes wrapping cycles in *X* and the circle, in the presence of strong *B*-field along *X*. The $(6+1)d$ non-commutative maximally supersymmetric *U*(*n*) gauge theory [18] on the D6 worldvolume leads at low-energy to quantum mechanics, with target the instanton moduli space ${\mathcal {M}}$. The K-theoretic DT partition function

4.1 $$\begin{aligned} Z^{7d}_{U(n)}(X) = \sum _{ch} e^{u(ch)} \int _{[{\mathcal {M}}_{ch}]^{virt}} e^{\omega +\mu _T} {\hat{A}}_T \end{aligned}$$

is the generating function obtained by integrating *A*-roof genus on some virtual cycle. We denote topological data $ch = ch (F)$ for some curvature *F*, and the classical factor

4.2 $$\begin{aligned} u (ch) = \int _X e^{\omega + H} \sqrt{{\hat{A}}(X)} ch (F) \end{aligned}$$

We denote *Z* the summation restricted to $ch_1(F)=0$ and ${\widehat{Z}}$ the unrestricted one. Integration is performed equivariantly with regard to a maximal torus *T* of $U(3)\times U(n)$, parametrized by $\Omega$-background parameters $q_1$, $q_2$, $q_3$ rotating *X* and Coulomb branch parameters $a_1,\ldots ,a_n$ acting on the D6 Chan–Paton indices.^6^ Each integral equals the twisted Witten index of the corresponding quantum mechanics. The BPS objects contributing to the index are D0, D2 and D4 branes, which wrap even-dimensional cycles in *X* and can bound to D6-branes. Localization reduces the computation to the fixed points of the action, which are in correspondence with plane partitions.

### Toric data

We review basic facts and fix notations. For $a=1,\ldots ,N$ and $i=1,\ldots ,n$, with $d = n-N>0$, take a matrix $Q_a^i$ with integer entries, and require that $\gcd (Q_a^1,\ldots ,Q_a^n)=1$ for all *a*. Let $t_a$ be positive real numbers. On ${{\mathbb {C}}}^n$ with coordinates $z_i$, define momentum maps ${{\mathbb {C}}}^n \rightarrow {{\mathbb {R}}}^N$

4.3 $$\begin{aligned} \mu _a(z) = \sum _i Q_a^i |z_i|^2 \end{aligned}$$

Consider the set $\mu ^{-1}(t) \subset {{\mathbb {C}}}^n$ and take the quotient by $U(1)^N$ acting as

4.4 $$\begin{aligned} z_i \rightarrow e^{\mathrm i\sum _a Q_a^i \alpha _a} z_i \end{aligned}$$

This is a subgroup of $U(1)^n$ acting as $z_i \rightarrow e^{\mathrm i\varepsilon _i }z_i$. The quotient is a *d*-dimensional toric variety *X*, on which $U(1)^d = U(1)^n/U(1)^N$ acts with moment maps $\mu _H$, which descend from

4.5 $$\begin{aligned} H = \sum _i \varepsilon _i |z_i|^2 \end{aligned}$$

Similarly, the Kahler form $\omega$ on *X* descends from the one on ${{\mathbb {C}}}^n$, and we have $\dim H_2(X) = N$. Geometrically, choose a basis $C_a$ of $H_2(X)$. The matrix $Q^i_a = D^i \cdot C_a$ represents intersection of toric divisors $D^i = \{z_i=0\}\cap X$ with curves $C_a$, and $t_a= \int _{C_a}\omega$. We are interested in $d=3$ and *X* Calabi–Yau, which implies $\sum _i Q_a^i = 0$.

To a toric threefold *X*, we can associate its polyhedron $\Delta _X$, given by the image of $\mu _H$. This has real dimension 3, and it is non-compact if *X* is non-compact. We call *vertices* its zero-dimensional faces, $v\in \Delta _X^{(0)}$, the fixed points of the $U(1)^3$ action discussed above. Every vertex has valence 3, namely there are 3 fixed lines (some of which can be non-compact) emanating from it. Restricting to the compact skeleton of $\Delta _X$, we call *edges* the one-dimensional faces, $e \in \Delta _X^{(1)}$, and *faces* the two-dimensional ones, $f \in \Delta _X^{(2)}$. Denote by $n_f$ the number of faces. Generically, the number of edges in $\Delta _X^{(1)}$ is larger than *N*.

Around each vertex $v \in \Delta _X^{(0)}$, we can choose local coordinates, made out of $U(1)^N$-invariant combinations of $z_i$ variables. These are acted upon by $U(1)^d$, their weights being the local $\Omega$-background parameters (aka twisted masses in the GLSM language), denoted by $q_1^{(v)}$, $q_2^{(v)}$, $q_3^{(v)}$ for $v \in \Delta _X^{(0)}$, with $q_a^{(v)} = e^{\beta \epsilon _a^{(v)}}$. They are functions of the global $\varepsilon$’s and transform in the same way as the local coordinates, so only one such set is independent: we denote it by $q_1$, $q_2$, $q_3$. There’s no canonical choice for such $q_1$, $q_2$, $q_3$. The CY condition reads $q_{123}:=q_1 q_2 q_3 =1$, but we do not need to impose it. We will often leave the label (*v*) implicit and denote $P_{123}=(1-q_1)(1-q_2)(1-q_3)$, $P_a=1-q_a$ for $a=1,2,3$.

For our gauge-theoretic purposes, we associate an integer $m_f$ to each $f \in \Delta _X^{(2)}$ (this integers correspond to $c_1(F)$ of the 6d curvature *F*.) From the viewpoint of a vertex, there are three such integers, associated to the three faces this vertex sees (with the understanding the $m=0$ for a non-compact face). Let

4.6 $$\begin{aligned} (q^{(v)})^m = (q_1^{(v)})^{m_{23}} (q_2^{(v)})^{m_{13}} (q_3^{(v)})^{m_{12}} = e ^{ \beta \epsilon ^{(v)} \cdot m} \end{aligned}$$

where we identify direction 1 with face along 23, etc. If $e \in \Delta _X^{(1)}$ connects vertices $v_1$ and $v_2$, then we have

4.7 $$\begin{aligned} \epsilon _\tau ^{(v_2)} = - \epsilon _\tau ^{(v_1)}, \quad \epsilon _{n_1}^{(v_2)} = \epsilon _{n_1}^{(v_1)} - \psi _{n_1}^{(e)} \epsilon _\tau ^{(v_1)}, \quad \epsilon _{n_2}^{(v_2)} = \epsilon _{n_2}^{(v_1)} - \psi _{n_2} ^{(e)}\epsilon _\tau ^{(v_1)} \end{aligned}$$

for some integers $\psi _{n_1}^{(e)}$ and $\psi _{n_2} ^{(e)}$. (Here $\tau$ is for tangent, $n_1$ and $n_2$ for normal directions to the edge.) In other words, $e \sim {{\mathbb {P}}}^1$ and its normal bundle in *X* splits as

4.8 $$\begin{aligned} {\mathcal {N}}= {\mathcal {O}}(-\psi _{n_1}^{(e)}) \oplus {\mathcal {O}}(-\psi _{n_2}^{(e)}) \end{aligned}$$

If *X* is CY, then $\psi _{n_1}^{(e)}+\psi _{n_2} ^{(e)}= -2$. We define

4.9 $$\begin{aligned} \psi ^{(e)} \cdot m = \sum _{v \in e} \frac{\epsilon ^{(v)} \cdot m}{\epsilon ^{(v)}_\tau } \end{aligned}$$

the sum being over the two vertices that belong to *e*. This equals

4.10 $$\begin{aligned} \psi ^{(e)} \cdot m = \psi ^{(e)}_{n_1} m_{n_1} + \psi ^{(e)}_{n_2} m_{n_2} + \sum _{v\in e} m_{\tau } \end{aligned}$$

Again, the sum is over the two vertices that belong to *e*, and $m_\tau$ refers to the face with normal direction $\tau$ at *v*. This is cumbersome (but well-defined), and we’ll make it more geometric in a moment. Given a Young diagram $\lambda$ (see below), we define

4.11 $$\begin{aligned} f_\lambda ^{(e)} = \sum _{(a,b) \in \lambda } \psi _{n_1}^{(e)} \left( a-\frac{1}{2} \right) + \psi _{n_2}^{(e)} \left( b-\frac{1}{2} \right) \end{aligned}$$

Denote by $t_e = \sum _{v \in e} \frac{H_v}{\epsilon _e^{(v)}}$ its size and $Q_e = e^{t_e}$.

#### From local to global

The work [44] studies a map^7^ from $H^2_{c} (X)$ to $H^2 (X)$

4.12 $$\begin{aligned} m = (m^i)_{i \in \Delta _X^{(2)}} \mapsto (\psi .m)^a := \sum _{i \in \Delta _X^{(2)}} Q_i^a m^i, \quad a=1,\ldots ,N \end{aligned}$$

In that context, the geometry behind Eqs. (4.6) and (4.9) is clear: they are local versions of the global map just defined. Borrowing certain definitions^8^ and results from there, we explain why this is the case.

Consider the K-equivariant integral^9^

4.14 $$\begin{aligned} Z_q (t) - Z_q (t+\psi .m) \prod _{i \in \Delta _X^{(2)}} q_i^{m^i} = \oint _{JK} d\phi \, e^{\phi .t} \frac{1-\prod _{i \in \Delta _X^{(2)}} e^{-\beta x_i m^i}}{\prod _{i=1}^n (1-e^{-\beta x_i})} \end{aligned}$$

The relation between Chern roots $x_i := \varepsilon _i + \sum _a Q_i^a \phi _a$ and local $\epsilon ^{(v)}_{1,2,3}$ at a fixed point $v \in \Delta _X^{(0)}$ is such that, at any JK pole, all $x_i$’s are zero, except for three (in this paper $d=3$), from which we can read off the local $\epsilon ^{(v)}$’s. Moreover, each $\epsilon ^{(v)}_{a=1,2,3}$ couples to the face $f \in \Delta _X^{(2)}$ touching *v* and with normal direction $a=1,2,3$, precisely as in Eq. (4.6). From this, it follows that Eq. (4.9) is induced by Eq. (4.12), as implicitly assumed below.

All these properties are explicitly checked in the examples below.

### Partitions

We can think of higher-dimensional partitions recursively. Start from a Young diagram: this is a collection $\lambda =(\ell _1,\ldots ,\ell _s)$ with $s \ge 1$ of positive integers $\ell _i$ such that $\ell _i \ge \ell _{i+1}$ for $i=1,\ldots ,s-1$, and we denote its size by $|\lambda |=\sum _{i=1}^s \ell _i$. Inclusion is defined as $\lambda \subseteq \lambda '$ iff $\ell _i \le \ell _i'$ for all *i*. The next step is a plane partition: this is a collection $\pi =(\lambda _1,\ldots ,\lambda _s)$ of Young diagrams $\lambda _i$ such that $\lambda _{i+1} \subseteq \lambda _i$. Inclusion is defined as $\pi \subseteq \pi '$ iff $\lambda _i \subseteq \lambda _i'$ for all *i*’s, and the size is $|\pi |=\sum _{k=1}^s |\lambda _k|$. Equivalently we can think of a plane partition $\pi$ as a collection of non-negative integers $\{ \pi _{i,j} \}$ indexed by integers $i,j \ge 1$ subject to the condition

4.15 $$\begin{aligned} \pi _{i,j} \ge \max ( \pi _{i+1,j}, \pi _{i,j+1}) \quad \forall i,j \end{aligned}$$

The size is $|\pi | = \sum _{i,j} \pi _{i,j}$. In this formulation, we can regard the plane partition $\pi$ as the subset of points $(a,b,c) \in {{\mathbb {Z}}}^3$, such that $a,b,c \ge 1$ and $c \le \pi _{a,b}$. Its character is

4.16 $$\begin{aligned} K_\pi (q_1, q_2, q_3)= \sum _{(a,b,c) \in \pi } q_1^{a-1} q_2^{b-1} q_3^{c-1} \end{aligned}$$

A colored plane partition $\vec \pi = (\pi _1,\ldots ,\pi _n)$ is a *n*-dimensional vector of plane partitions, where we call *n* the rank. With $K_i = K_{\pi _i}$, we define its character as

4.17 $$\begin{aligned} K = \sum _{i=1}^n a_i K_i (q_1, q_2, q_3) \end{aligned}$$

Its size is $|\vec \pi |=\sum _{i=1}^n |\pi _i|$. We define the dual $K^*$ of *K* by replacing $q_a$ with $q_a^{-1}=q_a^*$ for $a \in \{1,2,3\}$ and similarly for $a_i$. We will often identify a plane partition with its character.

#### Regularization

The partitions are allowed to have infinite size. In this case, it is better to think of a partition $\pi$ in terms of the associated monomial ideal $I_\pi \subset {{\mathbb {C}}}[q_1,q_2,q_3]$,

4.18 $$\begin{aligned} \pi = \{ (k_1,k_2,k_3) \in {{\mathbb {Z}}}^3_{>0} | \,\prod _{a=1}^3 q_a^{k_a-1} \not \in I_\pi \} \end{aligned}$$

The asymptotics of $\pi$ along direction *a* is given by

4.19 $$\begin{aligned} \lambda _a = \lim _{q_a \rightarrow 1} P_a \pi \end{aligned}$$

and depends on all three variables except $q_a$. The regularized partition is defined as

4.20 $$\begin{aligned} K_{reg} = K - \sum _{\alpha =1}^3 \frac{\lambda _\alpha }{P_\alpha } \end{aligned}$$

In analogy with partitions, we define the size of a Laurent polynomial ${\mathcal {P}}(q_1,q_2, q_3)$ as

4.21 $$\begin{aligned} |{\mathcal {P}}| = {\mathcal {P}}(1,1, 1) \end{aligned}$$

which can be negative.

#### Plethystic substitutions

A Laurent polynomial in the variables $q_a$ and $a_i$ is *movable* when it does not contain $\pm 1$ factors in the sum. The map ${\hat{a}}$ is defined on movable Laurent polynomials as

4.22 $$\begin{aligned} {\hat{a}} : \sum _i p_i M_i \mapsto \prod _i \left( M_i^{1/2}-M_i^{-1/2}\right) ^{-p_i} \end{aligned}$$

where $M_i$ are monomials with unit coefficient and $p_i$ integers.

### Vertex formalism

For generic *X*, fixed points are in one-to-one correspondence with collections *I* of *n*-tuples of (possibly infinite size) plane partitions, located at the vertices of $\Delta _X$:

4.23 $$\begin{aligned} I = \{ \pi _v = (\pi _{1,v},\ldots , \pi _{n,v}) \}_{v \in \Delta _X^{(0)}} \end{aligned}$$

Each $\pi _{i,v}$ is a plane partition, and the collection satisfies certain compatibility conditions: $\pi _{i,v_1}$ and $\pi _{i,v_2}$ must have the same asymptotics along edge *e*, whenever $v_1$ and $v_2$ belong to *e*.

With $K_{i,v} = K_{\pi _{i,v}}(q_1^v,q_2^v,q_3^v)$ for $v\in \Delta _X^{(0)}$, the virtual tangent space at *I* is

4.24 $$\begin{aligned} T_I = \sum _{v\in \Delta _X^{(0)}} -P_{123} {\mathcal {H}}_v {\mathcal {H}}^*_v - T_{pert} \end{aligned}$$

where we defined

4.25 $$\begin{aligned} {\mathcal {H}}_v = \sum _i \frac{a_i}{P_{123}} - a_i K_{i,v} \end{aligned}$$

and subtracted the (divergent) perturbative factor

4.26 $$\begin{aligned} T_{pert} = \sum _{v\in \Delta _X^{(0)}} -\frac{1}{P_{123}^*} \sum _{i,j} \frac{a_i}{a_j} \end{aligned}$$

We can rewrite this as

4.27 $$\begin{aligned} T_I = \sum _{i,j} \frac{a_i}{a_j} N_{ij} \end{aligned}$$

where we defined

4.28 $$\begin{aligned} N_{ij} = \sum _{v\in \Delta _X^{(0)}} K^*_{j,v} -q_{123} K_{i,v} -P_{123}K_{i,v} K_{j,v}^* \end{aligned}$$

Since the partitions can only grow along compact cycles, we know that $T_I$ is a Laurent polynomial, and we are allowed to apply the ${\hat{a}}$ functor to it. The partition function, aka twisted Witten index, takes the form

4.29 $$\begin{aligned} Z^{7d}_{U(n)} (X) = \sum _I {\hat{a}} (T_I) \, e^{u(I)} \end{aligned}$$

Let us redistribute [10, 45] the various parts, such that each one is manifestly finite.

#### No faces

Let us consider the case with no faces. By using the regularized expression $K_{reg}$, we can write

4.30 $$\begin{aligned} N_{ij} = \sum _{v\in \Delta _X^{(0)}} T_{v,ij} + \sum _{ v\in \Delta _X^{(0)}} \sum _\alpha t_{\alpha ,ij} \end{aligned}$$

where the first term contains regularized contributions and all other finite pieces

4.31 $$\begin{aligned} T_{v,ij}= & {} K_{j,v,reg}^* - q_{123} K_{i,v,reg} -P_{123} K_{i,v,reg}K_{j,v,reg}^* \nonumber \\&-P_{123} K_{i,v,reg} \sum _\alpha \frac{\lambda _{j,\alpha }^*}{P_\alpha ^*} -P_{123} K_{j,v,reg}^* \sum _\alpha \frac{\lambda _{i,\alpha }}{P_\alpha } \nonumber \\&-P_{123} \sum _{\alpha \ne \beta } \frac{\lambda _{i,\alpha }}{P_\alpha }\frac{\lambda _{j,\beta }^*}{P_\beta ^*} \end{aligned}$$

while the second term contains the infinite partitions,

4.32 $$\begin{aligned} t_{e,ij} = \frac{\lambda _{j,e}^*}{P_e^*} -q_{123} \frac{\lambda _{i,e}}{P_e} -P_{123} \frac{\lambda _{i,e}}{P_e}\frac{\lambda _{j,e}^*}{P_e^*} \end{aligned}$$

and it produces a finite term

4.33 $$\begin{aligned} T_{e,ij} = \sum _{v \in e} t_{e,ij} \end{aligned}$$

once we sum over the two vertices belonging to the edge. Both $T_v$ and $T_e$ are movable Laurent polynomials. (So we can apply plethystic to them.) We have

4.34 $$\begin{aligned} T_I = \sum _{i,j} \frac{a_i}{a_j} \sum _{v\in \Delta _X^{(0)}} T_{v,ij} + \sum _{e \in \Delta _X^{(1)}} T_{e,ij} \end{aligned}$$

We apply Duistermaat–Heckman theorem to compute $ch=(ch_0,ch_1,ch_2,ch_3)$

4.35 $$\begin{aligned} \begin{aligned} ch_3&= \sum _i \left( \sum _{v \in \Delta _X^{(0)}} \left( -\frac{\alpha _i^3}{3! \epsilon _1^{(v)} \epsilon _2^{(v)}\epsilon _3^{(v)}} + |K_{i,v,reg}|\right) \right. \\&\quad \left. - \sum _{e \in \Delta _X^{(1)}} f_{\lambda _{e,i}}- \sum _{e \in \Delta _X^{(1)}} |\lambda _{i,e}| \sum _{v \in e} \frac{\alpha _i}{\epsilon _e^{(v)}} \right) \\ ch_2&= - \sum _i \left( \sum _{v\in \Delta _X^{(0)}} \frac{\alpha _i^2 H_v}{2 \epsilon _1^{(v)} \epsilon _2^{(v)}\epsilon _3^{(v)}} + \sum _{e \in \Delta _X^{(1)}} |\lambda _{i,e}| t_e \right) \\ ch_1&= -\sum _i \sum _{v\in \Delta _X^{(0)}} \frac{\alpha _i H^2_v}{2 \epsilon _1^{(v)} \epsilon _2^{(v)}\epsilon _3^{(v)}} \\ ch_0&= - n \sum _{v \in \Delta _X^{(0)}} \frac{H^3_v}{3! \epsilon _1^{(v)} \epsilon _2^{(v)}\epsilon _3^{(v)}} \end{aligned} \end{aligned}$$

The last term in $ch_3$ is zero for the present case, but will contribute when we turn on fluxes. The quantum mechanical expression Eq. (4.2) is obtained by setting $\alpha =0$. We get^10^

4.36 $$\begin{aligned} u^{U(n)}_{K,\lambda } (g,t) = g ch_3 + ch_2 - n \frac{1}{24} c_2(X) \cdot t + \frac{ch_0}{g^2 - (n\epsilon /2)^2} \end{aligned}$$

where we denoted

4.37 $$\begin{aligned} \epsilon = \epsilon _1 + \epsilon _2+\epsilon _3 + 2\mathrm i\pi \end{aligned}$$

With $-p = e^{g}$, we split the sum over *I* as a sum over $\pi$’s with given asymptotics $\lambda$ (vertex)

4.38 $$\begin{aligned} V_{v,\lambda }= \sum _{\pi |\lambda } (-p)^{\sum _i |K_{i,v,reg}|} \prod _{i,j} {\hat{a}} (\frac{a_i}{a_j} T_{v,ij}) \end{aligned}$$

and a sum over asymptotics, with the simple (edge) functions

4.39 $$\begin{aligned} E_e (\lambda ) = (-p)^{-\sum _i f_{\lambda _{e,i}}} Q_e^{-\sum _i |\lambda _{i,e}|} \prod _{i,j} {\hat{a}} (\frac{a_i}{a_j} T_{e,ij}) \end{aligned}$$

At this stage, there’s no clear relation between the $n=1$ and $n>1$ cases, which depend in a complicated way on Coulomb branch parameters. We get

4.40 $$\begin{aligned} Z^{7d}_{U(n)} (X;p,t_e,a_i) = e^{-\frac{n}{24} c_2(X) \cdot t + \frac{ch_0}{g^2-(n\epsilon /2)^2}} \sum _\lambda \prod _{v\in \Delta _X^{(0)}} V_{v,\lambda } \prod _{e\in \Delta _X^{(1)}} E_e(\lambda _e) \end{aligned}$$

### Rank one vertex and GV/PT

Let *X* be a non-compact toric threefold. Up to a technical assumption, if we normalize the rank one vertex by the empty vertex, the individual dependence on $q_1$, $q_2$, $q_3$ goes away [13, Section 7.1.3] and the result only depends on their product. The overall factor in Eq. (4.40) is such that, for a geometry *X* engineering theory ${{{\mathcal {T}}}_{X}}$, we get exactly [46–48] the full 5d instanton partition function of ${{{\mathcal {T}}}_{X}}$ featuring in Eq. (3.2):

4.41 $$\begin{aligned} \frac{Z_{U(1)}^{7d}(X)}{\prod _{v \in \Delta _X^{(0)}} Z_{U(1)}^{7d}({{\mathbb {C}}}^3) } = Z^{5d}_{{{{\mathcal {T}}}_{X}}}({{\mathbb {C}}}^2) \end{aligned}$$

provided $\Omega$-background parameters on the two sides are properly identified.

## General Theory

In this section we develop the general higher rank theory. We first state our main assumption, and then work out its implications. We first deal with the simpler case of no D4-branes, and then add D4-branes wrapping the hypersurfaces of *X*, corresponding to the faces in $\Delta _X$. For both cases, we derive a 7d master formula where the partition function completely factorizes. For geometries admitting a geometric engineering limit, this factorization reproduces exactly the 5d master formula on the corresponding $A_n$ space. The focus here is on general results, while some examples are presented in the following section.

### Key assumption

We *assume* independence on Coulomb moduli in the instanton sector.^11^ Mathematically, this independence mirrors the independence of equivariant parameters in [13], which is related to compactness of the corresponding moduli spaces, but we take it as an experimental fact. Again, all we need is the toric Calabi–Yau fivefold, so we can work, for example, with *U*(*n*) theory on ${{\mathbb {P}}}^3$ (which is engineered [13] by taking a resolution of singularities of the total space of the direct sum of two ${{{\mathbb {Z}}}}_{n}$-quotient of the sum of two line bundles, i.e. ${{\mathcal {O}}}(-2) \oplus {{\mathcal {O}}}(-2)$). We also don’t need to be within the realm of the geometric engineering in the sense of [11], e.g. we can analyze the theory on the total space of the line bundle ${\mathcal {O}}(-3) \rightarrow {{\mathbb {P}}}^2$.

We performed several experimental checks of our assumption both in the zero-flux sector, and when $c_1(F) \ne 0$ (highly non-trivial).

Physically, this independence is the independence of the partition function of the $\Omega$-deformed five-dimensional ${{\mathcal {N}}}=1$ supersymmetric theory on $\widetilde{{{{\mathbb {C}}}}^{2}/{{{\mathbb {Z}}}}_{n}}$ fibered over $S^1$, on the Kähler moduli of the resolution. This is the usual argument of the *Q*-exactness of the appropriate components of the stress-energy tensor. This means that the DT partition function can depend on seven-dimensional Coulomb moduli only via an overall universal factor, which we suppress in the following.

### Factorizations

Using notations and conventions of Eqs. (4.31) and (4.33), recall that

5.1 $$\begin{aligned} N_{ij} = \sum _{v\in \Delta _X^{(0)}} T_{v,ij} + \sum _{e\in \Delta _X^{(1)}} T_{e,ij} \end{aligned}$$

Observe that $N_{ji} = - q_{123} N^*_{ij}$. With $N_i = N_{ii}$, write

5.2 $$\begin{aligned} \sum _{i,j} \frac{a_i}{a_j} N_{ij} = \sum _i N_i + \sum _{i<j} \frac{a_i}{a_j} N_{ij} + \frac{a_j}{a_i} N_{ji} \end{aligned}$$

Because of the assumption, we can take whatever choice of $a_i$, and taking $a_i=L^i$ and then sending $L \rightarrow \infty$ is particularly convenient.

Set $a_i = L^i$ and look at the limit $L \rightarrow \infty$. For any monomial *x*, we have

5.3 $$\begin{aligned} {\hat{a}} (-L^{j-i} x) =(L^{j-i}x)^{1/2} (1-L^{i-j}x^{-1}) \end{aligned}$$

With $i<j$, taking the conjugate of last term, we compute

5.4 $$\begin{aligned} \lim _{L\rightarrow \infty } {\hat{a}} ( \frac{a_j}{a_i} N_{ji} + \frac{a_j}{a_i} N_{ij}^*) = \prod _{v\in \Delta _X^{(0)}} q_{123}^{\frac{1}{2} |K_{j,v,reg}|-\frac{1}{2} |K_{i,v,reg}|} \prod _{e\in \Delta _X^{(1)}} q_{123} ^{\frac{1}{2} (f_{\lambda _{i,e}} - f_{\lambda _{j,e}})} \end{aligned}$$

where quadratic pieces in $T_v$ and $T_e$ cancel out, either in $N_{ij}$ or when combining it with $N_{ji}^*$. Therefore we have^12^ for $i<j$

5.5 $$\begin{aligned} \lim _{L \rightarrow \infty } {\hat{a}} ( \frac{a_j}{a_i} N_{ji} + \frac{a_i}{a_j} N_{ij}) = (-q_{123}^{\frac{1}{2}})^{|N_{ij}|} \end{aligned}$$

with

5.6 $$\begin{aligned} |N_{ij}| = \sum _{v\in \Delta _X^{(0)}} |K_{v,reg,j}|-|K_{v,reg,i}| + \sum _{e\in \Delta _X^{(1)}} f_{\lambda _{i,e}} - f_{\lambda _{j,e}} \end{aligned}$$

We can write

5.7 $$\begin{aligned} \prod _{i<j} (-q_{123}^{\frac{1}{2}})^{|N_{ij}|} = \prod _{i=1}^n (-q_{123}^{\frac{1}{2}})^{(-n-1+2i)(\sum _e f_{\lambda _{i,e}}-\sum _v |K_{i,v,reg}|)} \end{aligned}$$

This proves factorization along $A_{n-1}$ for any *X* without D4-branes: summing over fixed points

5.8 $$\begin{aligned} Z^{7d}_{U(n)} (X) = \sum _{K} e^{u(K)} {\hat{a}} (T) = \sum _K e^{u} \prod _i {\hat{a}} (N_i) \prod _{i<j} (-q_{123}^{\frac{1}{2}})^{|N_{ij}|} \end{aligned}$$

where we postpone the discussion of classical parts.

### Adding faces

If there are compact 4-cycles, denote fundamental quantities by ${\tilde{a}}$, ${\tilde{K}}$. Let $a_i = {\tilde{a}}_i q^{m_i}$ and

5.9 $$\begin{aligned} K_{i,v} =q^{-m_i} \left( {\tilde{K}}_{i,v} - \frac{1-q^{m_i}}{P_{123}} \right) \end{aligned}$$

where the fluxes *m* are $n \times n_f$ integers.^13^ The perturbative factor is

5.10 $$\begin{aligned} T_{pert} = -\sum _{v\in \Delta _X^{(0)}} \frac{1}{P^*_{123}} \sum _{i,j} \frac{{\tilde{a}}_i}{{\tilde{a}} _j} \end{aligned}$$

The difference of perturbative factors in the two variables

5.11 $$\begin{aligned} {\mathcal {P}}_m = \sum _{v\in \Delta _X^{(0)}} \frac{1-q^{m}}{P^*_{123}} \end{aligned}$$

is a Laurent polynomial (so we can take its plethystic) satisfying ${\mathcal {P}}_{-m}=-q_{123}{\mathcal {P}}_m^*$. We have

5.12 $$\begin{aligned} T = \sum _{i,j} \frac{a_i}{a_j} N_{ij} + \sum _{i,j} \frac{{\tilde{a}}_i}{{\tilde{a}}_j} {\mathcal {P}}_{m_{ij}} \end{aligned}$$

where $m_{ij} = m_i - m_j$ and we introduced the short notation

5.13 $$\begin{aligned} q^{m_{ij}} N_{ij} = \sum _{v\in \Delta _X^{(0)}} q^{m_{ij}} T_{v,ij} + \sum _{v\in \Delta _X^{(0)}} q^{m_{ij}} \sum _\alpha t_{\alpha ,ij} \end{aligned}$$

Summing over fixed points

5.14 $$\begin{aligned} {\widehat{Z}}^{7d}_{U(n)} (X) = \sum _{K,m} e^{u(K,m)} {\hat{a}} (T) \end{aligned}$$

Looking at Eq. (4.35), we observe that now $\alpha _i = {\tilde{\alpha }}_i + m_i \cdot \epsilon$ depends on fixed point data. The last term in $ch_3$ now contributes as

5.15 $$\begin{aligned} \sum _{e \in \Delta _X^{(1)}} |\lambda _{i,e}| \sum _{v \in e} \frac{\alpha _i}{\epsilon _e^{(v)}} = \sum _{e\in \Delta _X^{(1)}} \psi \cdot m_i |\lambda _{e,i}| \end{aligned}$$

Hence we get

5.16 $$\begin{aligned} u^{U(n)}_{K,\lambda ,m} (g,t) = g ch_3 + ch_2 - \frac{n}{24} c_2(X) \cdot t + \frac{ch_0}{g^2 - (n\epsilon /2)^2} \end{aligned}$$

### Classicalities

#### Details

Let us explain how to compute

5.17 $$\begin{aligned} u_0 = \int _X e^{\omega + \mathrm iB} \, ch(F) \wedge \Gamma _X \end{aligned}$$

At large radius and *B*-field, this expression gives the central charge of the bound state, with $ch(F) \wedge \Gamma _X$ being its RR charge. For our purposes, it’s enough to only keep two terms:

5.18 $$\begin{aligned} \Gamma _X \sim 1 + \frac{\beta ^2}{24} c_2(X) \end{aligned}$$

For non-compact *X*, we define $u_0$ equivariantly.

It is useful to recall that ${\mathcal {H}}$ defined in Eq. (4.25) encodes *ch*(*F*) at the fixed points

5.19 $$\begin{aligned} \sum _{v \in \Delta _X^{(0)}} P_{123} {\mathcal {H}}_v = {\text {tr}} e^{\beta \Phi } \end{aligned}$$

*in the instanton background*, with $\Phi$ the adjoint scalar. An application of Duistermaat–Heckman theorem then gives

5.20 $$\begin{aligned} u_0 = \sum _{q=0}^3 \sum _{v \in \Delta _X^{(0)}} \frac{H_v^q}{\beta ^q q!} \frac{1}{\prod _{a=1}^3 \epsilon _a^{(v)}} {\text {coeff}}_{3-q} P_{123} {\mathcal {H}} \, (1 + \frac{\beta ^2}{24} \sum _{1 \le a<b \le 3} \epsilon _a^{(v)} \epsilon _b^{(v)}) \end{aligned}$$

for any toric threefold, with ${\text {coeff}}_p$ the coefficient of $\beta ^p$ in the small-$\beta$ expansion. Recalling

5.21 $$\begin{aligned} P_{123} {\mathcal {H}}_v = \sum _i \left( a_i -P_{123} a_i K_{i,v,reg} - a_i \sum _\alpha \lambda _\alpha \frac{P_{123}}{P_\alpha } \right) \end{aligned}$$

and expanding, one arrives at the result Eq. (4.35). Explicitly:

5.22 $$\begin{aligned} u_0 = (ch_3 + ch_1 \cdot \Gamma _2) + \beta ^{-1} (ch_2 + \frac{n}{24} c_2 (X) \cdot t) + \beta ^{-2} ch_1 + \beta ^{-3} ch_0 \end{aligned}$$

where we defined

5.23 $$\begin{aligned} ch_1 \cdot \Gamma _2 = \frac{1}{24} \sum _{i=1}^n \sum _{v\in \Delta _X^{(0)}} \alpha _i \sum _{1 \le a<b \le 3} \frac{\epsilon _a^{(v)} \epsilon _b^{(v)} }{\epsilon _1^{(v)} \epsilon _2^{(v)} \epsilon _3^{(v)}} \end{aligned}$$

and

5.24 $$\begin{aligned} c_2 (X) \cdot t = \sum _{v \in \Delta _X^{(0)}} \frac{H_v}{\epsilon _1^{(v)} \epsilon _2^{(v)}\epsilon _3^{(v)}} \sum _{1 \le a<b \le 3} \epsilon _a^{(v)} \epsilon _b^{(v)} \end{aligned}$$

Since in the main discussion we are not paying attention to terms linear in $\sum _i m_i$, the terms $\Gamma _2 \cdot ch_1$ and $ch_1$ have been dropped there. The same applies to powers of $\beta$, which are recovered by quantizing $\omega$.

We can write all terms involving only $\alpha$ and *H* in Eqs. (4.35) and (5.22) as

5.25 $$\begin{aligned} \sum _{i=1}^n \sum _{v \in \Delta _X^{(0)}} \sum _{p=0}^3 \frac{(H_v/\beta )^p \alpha _i^{3-p}}{p!(3-p)! \epsilon _1^{(v)} \epsilon _2^{(v)}\epsilon _3^{(v)}} = \sum _{i=1}^n \sum _{v \in \Delta _X^{(0)}} \frac{(H_v/\beta + \alpha _i)^3}{3! \epsilon _1^{(v)} \epsilon _2^{(v)}\epsilon _3^{(v)}} \end{aligned}$$

where $\alpha _i = {\tilde{\alpha }}_i + m_i \cdot \epsilon ^{(v)}$, and *m* can be non-zero only if compact divisors are present. Luckily, Eq. (5.25) only contributes either terms proportional to powers of *m*, or terms proportional to powers of ${\tilde{\alpha }}$, but not mixed terms.^14^ The former can be computed (see the examples), while the latter can be discarded as overall constants, together with the perturbative part in ${\tilde{a}}$ variables. Incidentally, this is the only dependence on 7d Coulomb moduli left if we trust our working assumption.

Finally, we can turn on flat RR potentials $C_{p+1}^{RR}$ (*p* even) that couple to the RR charge, thus promoting $u_0$ to

5.26 $$\begin{aligned} u = \int _{S^1 \times X} \left( \frac{ds}{g_s} e^{\omega + \mathrm iB} + \mathrm i\sum _p C_{p+1}^{RR} \right) \, ch(F) \wedge \Gamma _X \end{aligned}$$

For *D*0-branes, this gives the complexified (dimensionless) quantity

5.27 $$\begin{aligned} g := \frac{\beta }{\ell _s g_s} + \mathrm i\int _{S^1} C_1^{RR} \end{aligned}$$

which multiplies $ch_3$ in Eq. (4.36), with $R \sim \ell _s g_s$ the TN-radius. For higher *Dp*-branes, it is unclear how to extend equivariantly $C^{RR}$ from first principles, so as to make Eq. (5.26) well-defined. However, Eq. (5.25) suggests a 5d fixed-point interpretation: the index *i* runs over 2d fixed points, and index *v* over 3d ones. If we replace our $\alpha _i$ with the 2d Hamiltonian $H_i$ on $TN_n$ space, and weigh it by the corresponding tangent weights $\epsilon _{4,5}^{(i)}$ at the fixed point,

5.28 $$\begin{aligned} \sum _{i,v} \frac{(H_v + \alpha _i)^3}{3! \epsilon _1^{(v)} \epsilon _2^{(v)} \epsilon _3^{(v)} } \rightarrow \sum _{i,v} \frac{(H_v + H_i)^3}{3! \epsilon _1^{(v)} \epsilon _2^{(v)} \epsilon _3^{(v)} \epsilon _4^{(i)} \epsilon _5^{(i)} } \end{aligned}$$

then we can read off the remaining coupling (the coupling for $ch_0$ in Eq. (4.36)) from Eq. (A.27): just interpret the $\alpha _i$’s of $A_n$ space as the $\alpha _i$’s of our gauge theory, neglecting the cubic adjoint terms (which will pop up again as the limit of the perturbative part). The obtained action Eq. (4.36) is a suitable candidate for the equivariant extension of Eq. (5.26), and it satisfies many non-trivial checks (see below), in particular factorizability.

#### Shift equations

The term $ch_0$ in Eq. (4.35) is problematic for non-compact *X*. Let us define

5.29 $$\begin{aligned} {\mathcal {F}} (t, \varepsilon ) = - \sum _{v \in \Delta _X^{(0)}} \frac{H^3_v}{3! \epsilon _1^{(v)} \epsilon _2^{(v)} \epsilon _3^{(v)}} \end{aligned}$$

with notations as in Sect. 4.2. This is a regularized triple intersection for *X*. We have evidence [44] that, when *X* has at least one compact four-cycle,

5.30 $$\begin{aligned} - \sum _{v \in \Delta _X^{(0)}} \frac{(H_v + \epsilon \cdot m)^3}{3! \epsilon _1^{(v)} \epsilon _2^{(v)}\epsilon _3^{(v)}} = {\mathcal {F}}(t,\varepsilon ) + {\mathcal {F}}_{shift}(t,m) \end{aligned}$$

where we used the way $\alpha$ is shifted in Eq. (5.25) and ${\mathcal {F}}_{shift}$ is a function of *t*, *m* independent of regulators. If we choose^15^ some of the $\varepsilon$ such that

5.31 $$\begin{aligned} - \sum _{v \in \Delta _X^{(0)}} \frac{(H_v + \epsilon \cdot m)^3}{3! \epsilon _1^{(v)} \epsilon _2^{(v)}\epsilon _3^{(v)}} = {\mathcal {F}}(t -\psi \cdot m,\varepsilon ) \end{aligned}$$

then we have

5.32 $$\begin{aligned} {\mathcal {F}}(t,\varepsilon )= {\mathcal {F}}(t-\psi \cdot m,\varepsilon ) - {\mathcal {F}}_{shift}(t,m) \end{aligned}$$

By also choosing *m* such that $t-\psi \cdot m=0$ (choosing $\dim H_4$ out of $\dim H_2$ *t* variables), we get a prescription to compute the regularized triple intersection as $-{\mathcal {F}}_{shift}(t,m)$ in terms of DH sums.

Likewise, if in Eq. (5.24) we set $\varepsilon$’s corresponding to compact divisors to zero, we can study the difference $c_2(X) \cdot (t + \psi \cdot m) - c_2(X) \cdot t$.

We spell this out for some examples in Sect. 6.

### 7d master formula

Let us enforce our Coulomb independence assumption. Setting ${\tilde{a}}_i = L^i$ and taking the large *L* limit with $i<j$, we get (this equality is proved momentarily in Sect. 5.5.1)

5.33 $$\begin{aligned} \lim _{L \rightarrow \infty } {\hat{a}} \left( \frac{a_j}{a_i} N_{ji} + \frac{a_i}{a_j} N_{ij} \right) = (-q_{123}^{\frac{1}{2}})^{s_{ij}} \end{aligned}$$

with the integer given by

5.34 $$\begin{aligned} s_{ij} = |N_{ij}| + \sum _{e\in \Delta _X^{(1)}} (|\lambda _{i,e}| + |\lambda _{j,e}|) (\psi \cdot m_{ij}) \end{aligned}$$

This proves factorization for ${\hat{a}}(N_i)$. Summing over fixed points, we get

5.35 $$\begin{aligned} {\widehat{Z}} = \sum _{m,K} e^u \prod _{i<j} \left( -q_{123}^{\frac{1}{2}}\right) ^{s_{ij}+|{\mathcal {P}}_{m_{ij}}|} \prod _{i=1}^n {\hat{a}} (N_i) \end{aligned}$$

Let $$m_* = \sum _{i=1}^n m_i$$ and

5.36 $$\begin{aligned} \sigma _\ell (m):= & {} \sum _{i=1}^{\ell -1} m_i - \sum ^n_{i=\ell +1} m_i = 2\sum _{i=1}^\ell m_i-m_\ell -\sum _{i=1}^n m_i \end{aligned}$$

5.37 $$\begin{aligned} g_i= & {} g + \frac{\epsilon }{2}(n+1-2i) \end{aligned}$$

Using results from Appendix C and some extra tools [44], one shows that

5.38 $$\begin{aligned}&u^{U(n)}_{K,\lambda ,m} (g,t) + \frac{\epsilon }{2} \sum _{i<j} (s_{ij} + |{\mathcal {P}}_{m_{ij}}|) \nonumber \\&\quad = \sum _i u^{U(1)}_{K_i,\lambda _i,0} \left( g_i,t + g_i \psi \cdot m_i+ \frac{\epsilon }{2} \psi \cdot \sigma _i \right) \mod m_* \end{aligned}$$

and arrives at the 7d master formula

5.39 $$\begin{aligned} \boxed { {\widehat{Z}}^{7d}_{U(n)} (X; p,Q_e) = \mathop \sum \limits _{m \in {{\mathbb {Z}}}^{n \times n_f}} e^{f(m_*)} \prod _{i=1}^n \mathop \sum \limits _{K_i,\lambda _i} e^{u^{U(1)}_{K_i,\lambda _i,0} \left( g_i,t + g_i \psi \cdot m_i+ \frac{\epsilon }{2} \psi \cdot \sigma _i \right) } \, {\hat{a}} (N_i) }\nonumber \\ \end{aligned}$$

where the partition function completely factorizes in each $m_*$ sector. The function of $f(m_*)$, which is computable in our formalism up to a term linear in $m_*$ coming from $c_1$, is a cubic polynomial in $m_*$ that goes to zero for $m_*=0$.

If we normalize by the empty vertex and use Eq. (4.41), we can match *exactly* the 5d gauge theory result Eq. (3.5) upon setting $m_*=0$, for any geometry *X* engineering theory ${{{\mathcal {T}}}_{X}}$, if in Eqs. (4.37) and (A.22) we identify

5.40 $$\begin{aligned} \epsilon _4 + \epsilon _5 = \epsilon , \quad n \frac{\epsilon _4-\epsilon _5}{2} = g \end{aligned}$$

It is amusing to observe that in these cases $n_f = {\text {rk}} {{{\mathcal {T}}}_{X}}$.

The map between $m_i$ and $h_i$ in Eq. (3.5) depends on the details of geometric engineering. Until now we denoted the dependence of $Z^{7d}$ on $t_e$, $e \in \Delta _X^{(1)}$ in order to have a clear interpretation of the various shifts. Before applying the geometric engineering dictionary and as discussed in Sect. 4.2, we expand $t_e$ in a basis of $H_2(X)$.

#### Proof

Let us prove Eq. (5.33). Setting ${\tilde{a}}_i = L^i$ we compute for $i<j$

5.41 $$\begin{aligned} \lim _{L \rightarrow \infty } {\hat{a}} (\frac{a_j}{a_i} N_{ji} + \frac{a_i}{a_j} N_{ij}) \end{aligned}$$

The first observation is that this is equal to

5.42 $$\begin{aligned} (-q_{123}^{\frac{1}{2}})^{|q^{m_{ij}} N_{ij}|} \end{aligned}$$

So we just need to compute its net size. The first term gives

5.43 $$\begin{aligned} \sum _{v \in \Delta _X^{(0)}} |K_{j,v,reg}| - |K_{i,v,reg}| \end{aligned}$$

The second term gets a contribution from $\lambda ^*_j$ and one from $-q_{123} \lambda _i$. The first one gives

5.44 $$\begin{aligned} \lim _{q_1 \rightarrow 1} \sum _{(a,b) \in \lambda _{e,j}} \frac{-1 + q_1^{-1-\psi \cdot m_{ij} +\psi _2(a-1)+\psi _3(b-1)}}{1-q_1} = \psi \cdot m_{ij} |\lambda _{e,j}| - f_{\lambda _{e,j}} \end{aligned}$$

where in intermediate steps we can take the edge along direction 1. The second one gives

5.45 $$\begin{aligned} \psi \cdot m_{ij} |\lambda _{e,i}| + f_{\lambda _{e,i}} \end{aligned}$$

Combining them we get the result.

## Experimental Evidence

We discuss some examples in detail, focusing on some of the simplest cases. Many more examples could be added. Our purpose here is to explain in detail notations and perform explicit checks of general results.

### *SU*(*N*) examples

With notations as in [51], the 5d SCFT giving the UV completion of 5d ${\mathcal {N}}=1$ $SU(N)_k$ gauge theory is obtained in M-theory on a singularity whose toric diagram has external points at

6.1 $$\begin{aligned} D_0 = (0, 0)\,, \quad D_N= (0,N)\,, \quad D_x= (-1, w_x)\,, \quad D_y=(1, w_y)\,, \end{aligned}$$

with $w_x, w_y \in {{\mathbb {Z}}}$. We impose the convexity condition

6.2 $$\begin{aligned} 0<w< 2N, \quad w\equiv w_x + w_y \end{aligned}$$

The Chern–Simons level is $k = w - N$. The toric divisors satisfy relations

6.3 $$\begin{aligned} \begin{aligned} D_0&\cong (N-1) D_N + (w-2)D_x+\sum _{a=1}^{N-1} (a-1) E_a\\ D_N&\cong - D_0 - 2 D_x - \sum _{a=1}^{N-1} E_a\\ D_x&\cong D_y. \end{aligned} \end{aligned}$$

**Fig. 1:**
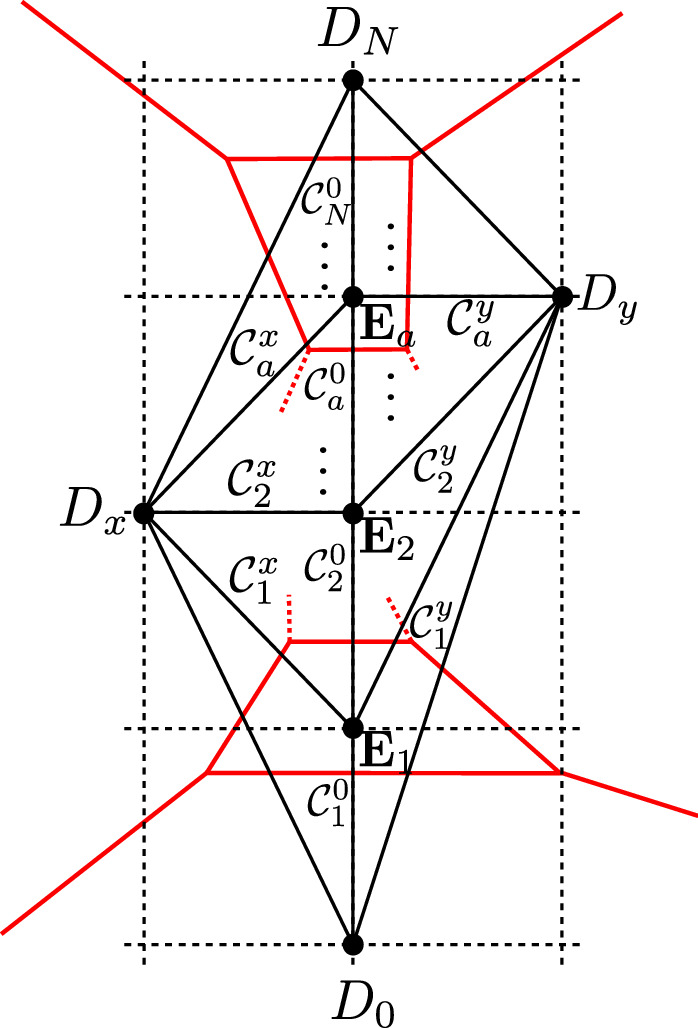
The $SU(N)_k$ gauge theory phase. We denote by $D_0$, $D_N$, $D_x$ and $D_y$ the non-compact divisors corresponding to external points in Eq. (6.1) and $E_1, \ldots , E_{N-1}$ the compact divisors, corresponding to internal points (0, *a*) for $a=1,\ldots ,N-1$

The resolution in Fig. 1 contains the curves

6.4 $$\begin{aligned}&C_a^x \cong D_x \cdot E_a, \quad C_a^y \cong D_y \cdot E_a, \quad a=1, \ldots N-1,\nonumber \\&C_a^0 \cong E_{a-1} \cdot E_a, \quad a= 1, \ldots N, \end{aligned}$$

where we denoted $D_0$ as $E_0$ and $D_N$ as $E_N$. One finds

6.5 $$\begin{aligned} C_a^0 - C_{a+1}^0 \cong (w-2 a) C_a^x, \quad a=1, \ldots , N-1. \end{aligned}$$

One can intersect the *N* independent curves $(C_1^0, C_a^x)$, whose volumes are $vol (C_1^0)=t_1$, $vol (C_a^x) = t_{a+1}$, with divisors $(D_0, E_b, D_N, D_x, D_y)$ to get the GLSM description

6.6 $$\begin{aligned} Q= \begin{pmatrix} w-2 &{} -w \delta _{1,b} &{} 0 &{} 1&{} 1 \\ \delta _{a,1}&{} -A_{ab} &{} \delta _{a, N-1} &{} 0&{} 0 \end{pmatrix} \end{aligned}$$

with $$a, b = 1, \ldots , N-1$$, and

6.7 $$\begin{aligned} A_{ab} = 2 \delta _{ab} - \delta _{a,b+1}-\delta _{a+1,b}. \end{aligned}$$

With notations as in Sect. 4.2, the toric variety obtained from symplectic quotient engineers *SU*(*N*) gauge theory with Chern–Simons level *k*. We can define

6.8 $$\begin{aligned} J = \mu _x D_x + \sum _{a=1}^{N-1} \nu _a E_a. \end{aligned}$$

The parameters $\nu _a = - \varphi _a$, $\mu _x = h$ are related to the FI parameters by

6.9 $$\begin{aligned} t_1 = h + (k+N) \varphi _1, \quad t_{a+1} = \sum _{b} A_{ab} \varphi _b \end{aligned}$$

Taking the cube one finds the field theory prepotential for $SU(N)_k$,

6.10 $$\begin{aligned} {\mathcal {F}} = - \frac{1}{6} J^3 = - \frac{1}{2} \mu _x^2 \nu _a (D_x^2 E^a) - \frac{1}{2} \mu _x \nu _a \nu _b (D_x E^a E^b) - \frac{1}{6} \nu _a \nu _b \nu _c (E^aE^bE^c)\nonumber \\ \end{aligned}$$

where we set^16^$D_x^3=0$. The non-zero triple-intersections are

6.11 $$\begin{aligned} D_x E_a E_b = - A_{ab}, \quad E_a^3=8, \quad E_{a-1}^2 E_a = w-2 a, \quad E_{a-1} E_a^2 = 2 a-2 -w.\nonumber \\ \end{aligned}$$

### The case of $SU(2)_0$

We consider the CY manifold $X={\mathcal {O}}(-2,-2) \rightarrow {{\mathbb {P}}}^1\times {{\mathbb {P}}}^1$, which corresponds to the 5d theory with *SU*(2) gauge group and zero Chern–Simons level.

The toric variety *X* can be constructed as the Kähler quotient of ${{\mathbb {C}}}^5$ by $U(1)^2$ with the action defined by the charge matrix

6.12 $$\begin{aligned} Q = \begin{pmatrix} 1 &{} 1 &{} 0 &{} 0 &{} -2 \\ 0 &{} 0 &{} 1 &{} 1 &{} -2 \end{pmatrix}, \end{aligned}$$

and moment maps

6.13 $$\begin{aligned} \begin{aligned} |z_1|^2 + |z_2|^2 - 2 |z_5|^2&= t_1,\\ |z_3|^2 + |z_4|^2 - 2 |z_5|^2&= t_2, \end{aligned} \end{aligned}$$

where we have assumed that $\{z_i\}, i=1,\ldots ,5$ parametrize ${{\mathbb {C}}}^5$. This toric manifold can be covered by 4 affine charts associated to the fixed points under the $T^3$-action. These charts can be parametrized with the set of coordinates that we summarize below in the table:6.14where the third column corresponds to the $T^3$-action at the corresponding fixed point written in terms of $\varepsilon _i$ that parametrize $T^5$ acting on ${{\mathbb {C}}}^5$. The last column corresponds to the value of the Hamiltonian $H= \sum _{i=1}^5 \varepsilon _i |z_i|^2$ at each fixed point. Alternatively we can parametrize the $T^3$-action in terms of three independent (global) $(\epsilon _1, \epsilon _2, \epsilon _3)$6.15If we denote by $H_v$ ($v=1,2,3,4$) the Hamiltonian at the fixed points we have

6.16 $$\begin{aligned} \begin{aligned} H_2 - H_1 = \epsilon _2 t_2 \\ H_3 - H_1 = \epsilon _1 t_1 \\ H_4 - H_3 = \epsilon _2 t_2 \\ H_4 - H_2 = \epsilon _1 t_1 \end{aligned} \end{aligned}$$

which are expressed in terms of global $(\epsilon _1, \epsilon _2, \epsilon _3)$. These shifts are uniquely fixed by the compact ${{\mathbb {P}}}^1$’s. The relevant geometry (vertices and edges) is conveniently summarized by 
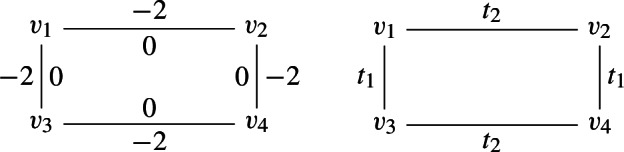
 where the first diagram keeps track of $\psi$ data, the second of edge sizes $t_e$. The geometry has one compact face, so $m \in {{\mathbb {Z}}}^n$, and Eq. (4.9) becomes for all four edges

6.18 $$\begin{aligned} \psi \cdot m_\ell = -2 m_\ell . \end{aligned}$$

Using this toric data we can perform the explicit calculations relevant for 7d theory on this geometry. The contribution of fluxes to the classical terms in Eq. (4.35) is

6.19 $$\begin{aligned} \begin{aligned} - \sum _i \sum _{v \in \Delta _X^{(0)}} \frac{({\tilde{\alpha }}_i + m_i \epsilon _3^{(v)})^3}{3! \epsilon _1^{(v)} \epsilon _2^{(v)}\epsilon _3^{(v)}}&= - \sum _i \sum _{v \in \Delta _X^{(0)}} \frac{{\tilde{\alpha }}_i^3}{3! \epsilon _1^{(v)} \epsilon _2^{(v)}\epsilon _3^{(v)}} - \frac{4}{3} \sum _i m_i^3, \\ - \sum _i \sum _{v\in \Delta _X^{(0)}} \frac{({\tilde{\alpha }}_i+ m_i \epsilon _3^{(v)})^2 H_v}{2 \epsilon _1^{(v)} \epsilon _2^{(v)}\epsilon _3^{(v)}}&= - \sum _i \sum _{v\in \Delta _X^{(0)}} \frac{{\tilde{\alpha }}_i^2 H_v}{2 \epsilon _1^{(v)} \epsilon _2^{(v)}\epsilon _3^{(v)}} - (t_1 + t_2) \sum _i m_i^2, \\ -\sum _i \sum _{v\in \Delta _X^{(0)}} \frac{({\tilde{\alpha }}_i + m_i \epsilon _3^{(v)})H^2_v}{2 \epsilon _1^{(v)} \epsilon _2^{(v)}\epsilon _3^{(v)}}&= -\sum _i \sum _{v\in \Delta _X^{(0)}} \frac{{\tilde{\alpha }}_i H^2_v}{2 \epsilon _1^{(v)} \epsilon _2^{(v)}\epsilon _3^{(v)}} - t_1 t_2 \sum _i m_i, \end{aligned}\nonumber \\ \end{aligned}$$

where we used Eqs. (6.15) and (6.16). The classical action (with ${\tilde{\alpha }}=0$) is built out of

6.20 $$\begin{aligned} \begin{aligned} ch_3&= -\frac{4}{3} \sum _i m_i^3 + \sum _i \left( \sum _v |K_{i,v}^{reg}| - \sum _e f_{\lambda _{e,i}} \right) - \sum _{i,e} \psi \cdot m_i |\lambda _{e,i}|, \\ ch_2&= -(t_1+t_2) \sum _i m_i^2 -\sum _{i,e} t_e |\lambda _{i,e}| ,\\ ch_1&= -t_1 t_2 \sum _i m_i ,\\ ch_0&= n {\mathcal {F}}(t), \end{aligned} \end{aligned}$$

where the last term requires a separate discussion. In Eq. (5.29) we define ${{\mathcal {F}}}(t, \varepsilon )$. Using the explicit toric data from Eq. (6.14) and setting $\varepsilon _5=0$ in Eq. (5.29) we get

6.21 $$\begin{aligned} {{\mathcal {F}}}(t_1, t_2 , \varepsilon ) = \frac{1}{12} t_2^2 (-3 t_1 + t_2) + f(\varepsilon ) (t_1 - t_2)^3, \end{aligned}$$

where

6.22 $$\begin{aligned} f (\varepsilon )= \frac{ \varepsilon _2^2 \varepsilon _3 \varepsilon _4 + \varepsilon _1^2 (\varepsilon _2 + \varepsilon _3) (\varepsilon _2 + \varepsilon _4) + \varepsilon _1 \varepsilon _2 (\varepsilon _3 \varepsilon _4 + \varepsilon _2 (\varepsilon _3 + \varepsilon _4))}{12 (\varepsilon _1 + \varepsilon _3) (\varepsilon _2 + \varepsilon _3) (\varepsilon _1 + \varepsilon _4) (\varepsilon _2 + \varepsilon _4)}. \end{aligned}$$

The ${{\mathcal {F}}}(t_1, t_2 , \varepsilon )$ has the property

6.23 $$\begin{aligned} {{\mathcal {F}}} (t_1 + 2m, t_2 + 2m, \varepsilon ) = {{\mathcal {F}}} (t_1, t_2,\varepsilon ) - m t_1 t_2 - m^2 (t_1 + t_2) - \frac{4}{3} m^3, \end{aligned}$$

where terms in *m* coincide with terms from Eq. (6.19). We extract the universal part

6.24 $$\begin{aligned} {\mathcal {F}} (t_1,t_2) = \frac{1}{12} t_2^2 (-3 t_1 + t_2), \end{aligned}$$

but we stress that we can also use ${{\mathcal {F}}}(t_1, t_2 , \varepsilon )$ from Eq. (6.21) since in what follows we only use the shift symmetry Eq. (6.23).

Finally let us compute the polynomial ${\mathcal {P}}$, defined in Eq. (5.11), for this example:

6.25 $$\begin{aligned} q_{123}^* {\mathcal {P}}_m (q_1, q_2, q_3)= & {} \frac{q_3^m -1}{(1-q_1)(1-q_2)(1-q_3)} + \frac{q_3^m q_2^{2m}-1}{(1-q_1)(1-q^{-1}_2)(1-q_3q_2^2)} \nonumber \\&+ \frac{q_3^m q_1^{2m}-1}{(1-q^{-1}_1)(1-q_2)(1-q_3q_1^2)} \nonumber \\&+ \frac{q_3^m q_1^{2m} q_2^{2m}-1}{(1-q^{-1}_1)(1-q^{-1}_2)(1-q_3 q_1^2 q_2^2)}. \end{aligned}$$

For $m>0$ we get

6.26 $$\begin{aligned} q_{123}^* {\mathcal {P}}_m (q_1, q_2, q_3) = - \sum _{s=0}^{m-1} \sum _{l=0}^{2s} \sum _{k=0}^{2s} q_3^s q_1^l q_2^k. \end{aligned}$$

Using the standard identities

6.27 $$\begin{aligned} \sum _{s=1}^n s = \frac{n(n+1)}{2},\quad \sum _{s=1}^{n} s^2 = \frac{n(n+1) (2n+1)}{6} \end{aligned}$$

we get

6.28 $$\begin{aligned} |{\mathcal {P}}_m| = {\mathcal {P}}_m (1, 1, 1) = - \sum _{s=0}^{m-1} (2s+1)^2 = \frac{1}{3} \left( m- 4 m^3 \right) , \end{aligned}$$

which is an integer, as expected. For $m<0$ we use the property

6.29 $$\begin{aligned} {\mathcal {P}}_{-m} (q_1, q_2, q_3) = - q^{-1}_{123} {\mathcal {P}}_m (q^{-1}_1, q^{-1}_2, q^{-1}_3), \end{aligned}$$

which implies

6.30 $$\begin{aligned} |{\mathcal {P}}_{-m}| = - |{\mathcal {P}}_m|, \end{aligned}$$

so it is clear that $|{\mathcal {P}}_m|$ is an odd function of *m*.

Using identities from Appendix C, we have

6.31 $$\begin{aligned} \sum _{i=1}^n \frac{{\mathcal {F}}(t + \psi \cdot g_i m_i+\frac{\epsilon }{2} \psi \cdot \sigma _i)}{g_i^2 - (\epsilon /2)^2}= & {} \frac{4}{3} \left( \frac{\epsilon }{2} \sum _{i<j} m_{ij}^3 +\frac{\epsilon ^2 g}{4g^2-(n\epsilon )^2} m_*^3 +g \sum _i m_i^3 \right) \nonumber \\&+\frac{n {\mathcal {F}}(t)}{g^2 - (n \epsilon /2)^2} \nonumber \\&-(t_1+t_2) \left( \frac{n \epsilon ^2}{4g^2-(n\epsilon )^2} m_*^2 +\sum _i m_i^2 \right) \nonumber \\&+t_1t_2 \frac{g m_*}{g^2 - (n \epsilon /2)^2} \end{aligned}$$

Alternatively, we can write it as

6.32 $$\begin{aligned}&\sum _{i=1}^n \frac{{\mathcal {F}}(t + \psi \cdot g_i m_i+\frac{\epsilon }{2} \psi \cdot \sigma _i)}{g_i^2 - (\epsilon /2)^2}\nonumber \\&\quad = \frac{\epsilon }{2} \frac{4}{3} \sum _{i<j} m_{ij}^3 + \frac{n {\mathcal {F}}(t + \psi \cdot g \frac{m_*}{n})}{g^2 - (n \epsilon /2)^2} + \frac{4}{3} g \sum _i \left( m_i - \frac{m_*}{n} \right) ^3 \nonumber \\&\qquad - \left( (t_1 + \psi \cdot g \frac{m_*}{n}) + (t_2 + \psi \cdot g \frac{m_*}{n}) \right) \sum _i \left( m_i - \frac{m_*}{n} \right) ^2. \end{aligned}$$

The first term in RHS of Eq. (6.31) comes from $|{\mathcal {P}}_m|=\frac{1}{3}(m-4 m^3)$, while the other term in ${\mathcal {P}}$ combines with $c_2(X)$. Indeed, using our prescription Eq. (5.24) for $c_2(X)\cdot t$, we can write the factorization formulas for the classical action: up to terms proportional to $m_*$, we get

6.33 $$\begin{aligned} u + \frac{\epsilon }{2} \sum _{i<j} (s_{ij} + |{\mathcal {P}}_{m_{ij}}|)= & {} \sum _i \frac{{\mathcal {F}}(t + g_i \psi \cdot m_i +\frac{\epsilon }{2} \psi \cdot \sigma _i)}{g^2_i - (\epsilon /2)^2} \nonumber \\&+\sum _i \left[ g_i \left( \sum _v |K_{i,v}^{reg}|-\sum _e f_{\lambda _{i,e}} \right) \right. \nonumber \\&\left. - \sum _{e} |\lambda _{i,e}| (t_e + g_i \psi \cdot m_i+\psi \cdot \sigma _i \frac{\epsilon }{2}) \right] \nonumber \\&- \frac{1}{24} c_2(X)\cdot (nt + 2 \frac{\epsilon }{2} \sum _{i<j} \psi \cdot m_{ij}) \end{aligned}$$

in agreement with Eq. (5.38). We use the property

6.34 $$\begin{aligned} -\frac{1}{24} c_2(X) \cdot (t + \psi \cdot m) + \frac{1}{24} c_2(X) \cdot t = \frac{m}{6} \end{aligned}$$

which can be checked explicitly from Eq. (5.24).

Finally we use the geometric engineering dictionary for *SU*(2) theory where Kähler parameters $(t_1, t_2)$ are related to the scalar $\varphi$ in *SU*(2) vector multiplet and the coupling *h* as

6.35 $$\begin{aligned} t_1 = h + 2\varphi , \quad t_2 = 2 \varphi . \end{aligned}$$

We then match *exactly* the 7d and 5d master formulas Eqs. (3.5) and (5.39) by identifying

6.36 $$\begin{aligned} h_{\ell } = -\sum _{i=1}^\ell m_i + \frac{1}{2} m_* = \frac{1}{2} \left( -m_1-\cdots -m_\ell +m_{\ell +1} + \cdots + m_n \right) \end{aligned}$$

and imposing the condition $m_* = 0$, which implies $h_0=h_n=0$ and amounts to going from *U*(*n*) to *SU*(*n*) in 7d. We conclude that the partition function for the 7d *SU*(*n*) theory on $X={\mathcal {O}}(-2,-2) \rightarrow {{\mathbb {P}}}^1\times {{\mathbb {P}}}^1$ is the same as the partition function for the 5d $SU(2)_0$ theory on $A_{n-1}$ space (both theories are extended to $S^1$ in the appropriate fashion). The classical part Eq. (6.24) becomes

6.37 $$\begin{aligned} {{\mathcal {F}}} (\phi , h) = - h \varphi ^2 - \frac{4}{3} \varphi ^3, \end{aligned}$$

as it should be. If instead we use Eq. (6.21), then we have

6.38 $$\begin{aligned} {{\mathcal {F}}} (\phi , h) = - h \varphi ^2 - \frac{4}{3} \varphi ^3 + f(\varepsilon ) h^3, \end{aligned}$$

which may correspond to adding some non-dynamical (purely geometric) term on the 5d side.

### A rank two example: $SU(3)_0$

Next we consider another example of CY that corresponds to *SU*(3) 5d gauge theory with zero Chern–Simons level. This CY can be obtained by the Käher quotient of ${{\mathbb {C}}}^6$ by $U(1)^3$ with action defined by the charge matrix

6.39 $$\begin{aligned} Q = \begin{pmatrix} 1 &{} 1 &{} 1 &{} -3 &{} 0 &{} 0 \\ 0 &{} 0 &{} 1 &{} -2 &{} 1 &{} 0 \\ 0 &{} 0 &{} 0 &{} 1 &{} -2 &{} 1 \end{pmatrix} \end{aligned}$$

and moment maps

6.40 $$\begin{aligned} \begin{aligned} |z_1|^2 + |z_2|^2 + |z_3|^2 - 3 |z_4|^2 = t_1\\ |z_3|^2 - 2|z_4|^2 + |z_5|^2 = t_2\\ |z_4|^2 - 2 |z_5|^2 + |z_6|^2 = t_3 \end{aligned} \end{aligned}$$

where we use ${{\mathbb {C}}}^6$ coordinates. The resulting manifold can be covered by 6 affine chats associated to the fixed points of $T^3$ action. We summarize this in the following tables:6.41where in middle column we define the coordinates in every chart and in the right column we write the value of Hamiltonian $H=\sum _{i=1}^6 \varepsilon _i |z_i|^2$ at the corresponding fixed point. The $T^3$ action at every fixed point can be summarized in the following table6.42where we use ${{\mathbb {C}}}^6$ parameters. Equivalently we can rewrite it in terms of 3-independent parameters $(\epsilon _1, \epsilon _2, \epsilon _3)$6.43If we denote by $H_v$ the value of the Hamiltonian at the fixed point *v* then the difference of Hamiltonians reads

**Fig. 2:**
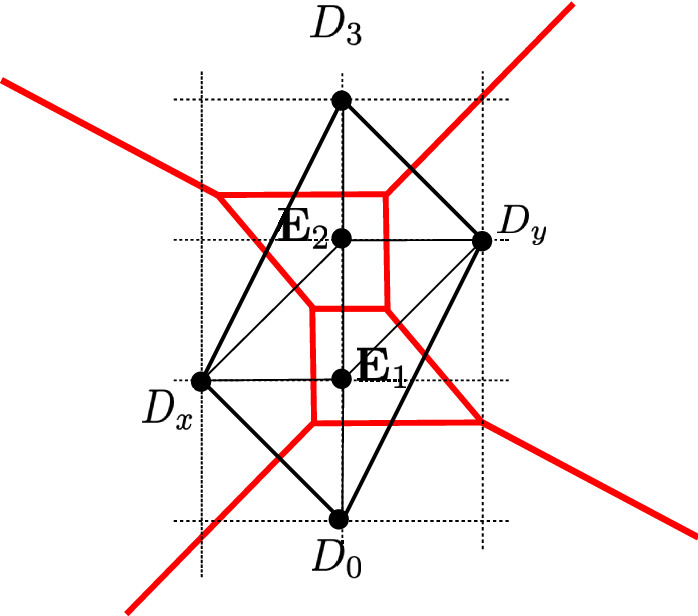
The $$SU(3)_0$$ geometry


6.44 $$\begin{aligned} \begin{aligned} H_2 - H_1&= \epsilon _3 t_1 \\ H_3 - H_1&= \epsilon _2 t_2 \\ H_4 - H_2&= (\epsilon _2 - \epsilon _3) t_2 \\ H_4 - H_3&= \epsilon _3 (t_1 - t_2) \\ H_5 - H_3&= (\epsilon _1 + 2 \epsilon _2) t_3 \\ H_6 - H_5&= \epsilon _3 (t_3 + t_1 - t_2) \\ H_6 - H_4&= (2\epsilon _2 + \epsilon _3 + \epsilon _1) t_3 \end{aligned} \end{aligned}$$

and this data is uniquely fixed by the compact part of the geometry. The relevant toric data (Fig. 2) can be encoded in the following pictures

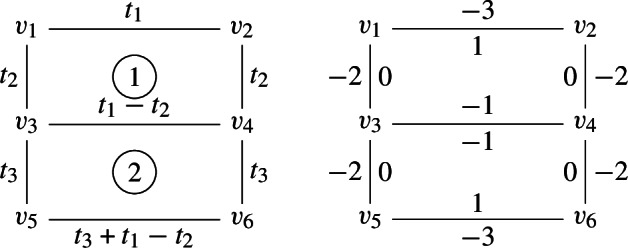
where the first diagram labels vertices, edges and faces together with sizes of the edges in terms moment map data, while the second diagram keeps track of $\psi$ data. Here we assume that $t_1-t_2>0$. The geometry has two compact faces (labeled by circles), so $m \in {{\mathbb {Z}}}^{2n}$, and Eq. (4.9) becomes6.46We have the following shifts of $\alpha$’s at each vertex

6.47 $$\begin{aligned} \begin{aligned} \alpha ^{(1)}&= \tilde{\alpha } + m_1 \epsilon _1 \\ \alpha ^{(2)}&= \tilde{\alpha } + m_1 (3 \epsilon _3 + \epsilon _1) \\ \alpha ^{(3)}&= \tilde{\alpha } + m_1 (\epsilon _1 + 2 \epsilon _2) - m_2 \epsilon _2 \\ \alpha ^{(4)}&= \tilde{\alpha } + m_1 ( 2\epsilon _2 + \epsilon _3 + \epsilon _1) + m_2 (\epsilon _3 - \epsilon _2) \\ \alpha ^{(5)}&= \tilde{\alpha } + m_2 (2\epsilon _1 + 3 \epsilon _2) \\ \alpha ^{(6)}&= \tilde{\alpha } + m_2 ( 2\epsilon _1 + 3 \epsilon _3 + 3 \epsilon _2) \end{aligned} \end{aligned}$$

where we suppressed the Lie algebra index for *U*(*n*) gauge theory. The contribution of fluxes to classical terms can be computed using only Eqs. (6.43) and (6.44):

6.48 $$\begin{aligned}&- \sum _i \sum _{v \in \Delta _X^{(0)}} \frac{(\alpha _i^{(v)})^3}{3! \epsilon _1^{(v)} \epsilon _2^{(v)}\epsilon _3^{(v)}} = \nonumber \\&\quad - \sum _i \sum _{v \in \Delta _X^{(0)}} \frac{{\tilde{\alpha }}_i^3}{3! \epsilon _1^{(v)} \epsilon _2^{(v)}\epsilon _3^{(v)}}- \sum _i \left( \frac{4}{3} m_{1,i}^3 + \frac{4}{3}m_{2,i}^3 - \frac{1}{2} m_{1,i}^2 m_{2,i} -\frac{1}{2} m_{1,i} m_{2,i}^2 \right) , \nonumber \\&\quad -\sum _i \sum _{v\in \Delta _X^{(0)}} \frac{\alpha _i^{(v)}H^2_v}{2 \epsilon _1^{(v)} \epsilon _2^{(v)}\epsilon _3^{(v)}} =-\sum _i \sum _{v\in \Delta _X^{(0)}} \frac{{\tilde{\alpha }}_i H^2_v}{2 \epsilon _1^{(v)} \epsilon _2^{(v)}\epsilon _3^{(v)}} \nonumber \\&\quad -\left( t_1 t_2- \frac{1}{2}t_2^2 \right) \sum _i m_{1,i} -\left( (t_1- t_2) t_3 + \frac{1}{2}t_3^2 \right) \sum _i m_{2,i}, \nonumber \\&\quad - \sum _i \sum _{v\in \Delta _X^{(0)}} \frac{(\alpha _i^{(v)})^2 H_v}{2 \epsilon _1^{(v)} \epsilon _2^{(v)}\epsilon _3^{(v)}} = - \sum _i \sum _{v\in \Delta _X^{(0)}} \frac{{\tilde{\alpha }}_i^2 H_v}{2 \epsilon _1^{(v)} \epsilon _2^{(v)}\epsilon _3^{(v)}} \nonumber \\&\quad - \frac{1}{2} \left( 2t_1 + t_2 \right) \sum _i m_{1,i}^2 -\left( t_2 - t_1 \right) \sum _i m_{1,i} m_{2,i} \nonumber \\&\quad - \frac{1}{2} \left( 3 t_3 + 2 t_1 - 2 t_2 \right) \sum _i m_{2,i}^2. \end{aligned}$$

If we set $\varepsilon _4=\varepsilon _5=0$ in Eq. (5.29) and perform the explicit computation

6.49 $$\begin{aligned} {{\mathcal {F}}} (t_1,t_2,t_3, \varepsilon )= & {} -\frac{1}{3} t_1(t_2^2+t_2 t_3 + t_3^2) +\frac{1}{6} t_2(2t^2_2 + 2t_2 t_3 + 3t_3^2) \nonumber \\&+ f(\varepsilon ) (t_1-2t_2 -t_3)^3, \end{aligned}$$

where

6.50 $$\begin{aligned} f(\varepsilon )= \frac{\varepsilon _2^2 \varepsilon _3 \varepsilon _6 + \varepsilon _1^2 (\varepsilon _2 + \varepsilon _3) (\varepsilon _2 + \varepsilon _6) + \varepsilon _1 \varepsilon _2 (\varepsilon _3 \varepsilon _6 + \varepsilon _2 (\varepsilon _3 + \varepsilon _6))}{18 (\varepsilon _6 + \varepsilon _1) (\varepsilon _6 + \varepsilon _2) (\varepsilon _1 + \varepsilon _3) (\varepsilon _2 + \varepsilon _3) } \end{aligned}$$

As expected the function ${{\mathcal {F}}} (t_1,t_2,t_3, \varepsilon )$ satisfies

6.51 $$\begin{aligned}&{{\mathcal {F}}} (t_1 +3m_1, t_2 - m_2 + 2m_1, t_3 - m_1 + 2m_2, \varepsilon ) \nonumber \\&\quad = {{\mathcal {F}}} (t_1, t_2, t_3, \varepsilon ) +\left( -\frac{4}{3} m_1^3 - \frac{4}{3} m_2^3 + \frac{1}{2} m_1^2 m_2 + \frac{1}{2} m_1 m_2^2 \right) \nonumber \\&\qquad + \left( m_1^2 (t_1 + \frac{1}{2} t_2) + m_2^2 (t_1 - t_2 + \frac{3}{2} t_3) + m_1 m_2 (t_2- t_1) \right) \nonumber \\&\qquad +\left( m_1 (-t_1 t_2 + \frac{1}{2} t_2^2) + m_2 (t_2 t_3 - t_1 t_3 - \frac{1}{2} t_3^2) \right) \end{aligned}$$

to be compared with Eq. (6.48). We focus on the universal part

6.52 $$\begin{aligned} {\mathcal {F}} (t_1, t_2, t_3) = -\frac{t_1 t_2^2}{3} + \frac{t_2^3}{3} - \frac{t_1 t_2 t_3}{3} + \frac{t_2^2 t_3}{2} - \frac{t_1 t_3^2}{3} + \frac{t_2 t_3^2}{2}, \end{aligned}$$

although in what follows we only use the shift symmetry Eq. (6.51). Finally we calculate ${\mathcal {P}}$ in an analogous way to the *SU*(2) case. For the given CY ${\mathcal {P}}$ is defined as

6.53 $$\begin{aligned} q_{123}^* {\mathcal {P}}_{(m_1,m_2)} (q_1, q_2, q_3)= & {} \frac{q^{m_1}_1 -1}{(1-q_1) (1-q_2) (1-q_3)} \nonumber \\&+ \frac{q_3^{3m_1} q_1^{m_1}-1}{(1-q_3^3q_1) (1-q_2 q_3^{-1}) (1-q_3^{-1})} \nonumber \\&+ \frac{q_1^{m_1} q_2^{2m_1} q_2^{-m_2} -1}{(1-q_2^{-1})(1-q_1 q_2^2)(1-q_3)}\nonumber \\&+ \frac{q_2^{2m_1} q_3^{m_1} q_1^{m_1} q_3^{m_2} q_2^{-m_2} -1}{(1-q_3 q_2^{-1})(1-q_2^2 q_3 q_1)(1-q_3^{-1})} \nonumber \\&+ \frac{q_1^{2m_2} q_2^{3m_2} -1}{(1-q_1^{-1} q_2^{-2})(1-q_1^2 q_2^3)(1-q_3)} \nonumber \\&+ \frac{q_1^{2m_2} q_3^{3m_2} q_2^{3m_2}-1}{(1-q_2^{-2} q_3^{-1} q_1^{-1})(1-q_1^2 q_3^3 q_2^3)(1-q_3^{-1})} \end{aligned}$$

which has the property

6.54 $$\begin{aligned} {\mathcal {P}}_{(-m_1,-m_2)} (q_1, q_2, q_3) = -q_{123}^{-1} {\mathcal {P}}_{(m_1,m_2)} (q^{-1}_1, q^{-1}_2, q^{-1}_3) \end{aligned}$$

Assuming $m_1 >0$ and $m_2 >0$ we can compute

6.55 $$\begin{aligned} \frac{ {\mathcal {P}}_{(m_1,m_2)} (q_1, q_2, q_3)}{q_{123}}= & {} - \sum _{s=0}^{m_1-1} \sum _{k=0}^{2s} \sum _{l=0}^{3s-k} q_1^s q_2^k q_3^l - \sum _{s=0}^{m_2-1} \sum _{k=0}^{2s} \sum _{l=0}^{s+k} q_1^k q_2^{2k-s} q_3^l \nonumber \\&+ \sum _{s=0}^{m_1-1} \sum _{l=0}^{m_2-1} \sum _{k=0}^{s+l} q_1^s q_2^{2s-l} q_3^k \end{aligned}$$

and its size

6.56 $$\begin{aligned} |{\mathcal {P}}_{(m_1,m_2)}| =- \frac{1}{3} \left( 4 m_1^3 - m_1 \right) - \frac{1}{3} \left( 4 m_2^3 - m_2 \right) + \frac{1}{2} m_1^2 m_2 + \frac{1}{2} m_1 m_2^2 \end{aligned}$$

for any integer $m_1$ and $m_2$.

Using the shift property Eq. (6.51) and formulas from Appendix C we can write

6.57 $$\begin{aligned} \sum _{i=1}^n \frac{{\mathcal {F}}(t + \psi \cdot g_i m_i+\frac{\epsilon }{2} \psi \cdot \sigma _i)}{g_i^2 - (\epsilon /2)^2}= & {} \frac{n {\mathcal {F}}(t + \psi \cdot g \frac{m_*}{n})}{g^2 - (n \epsilon /2)^2} +\frac{\epsilon }{2} \sum _{i<j} p(m_{1,ij}, m_{2,ij}) \nonumber \\&+ g \sum _i \left( p(m_{1,i}, m_{2,i}) -p(\frac{m_{1*}}{n}, \frac{m_{2*}}{n}) \right) \nonumber \\&+ \sum _i \left( (m_{1,i}^2- \frac{m_{1*}^2}{n^2}) (t_1 + \frac{1}{2} t_2) \right. \nonumber \\&+ (m_{2,i}^2 - \frac{m_{2*}^2}{n^2}) (t_1 - t_2 + \frac{3}{2} t_3) \nonumber \\&\left. + (m_{1,i} m_{2,i}- \frac{m_{1*}m_{2*}}{n^2}) (t_2- t_1) \right) , \end{aligned}$$

where we defined

6.58 $$\begin{aligned} p(m_1, m_2) = \frac{4}{3} m_1^3 + \frac{4}{3} m_2^3 - \frac{1}{2} m_1^2 m_2 - \frac{1}{2} m_1 m_2^2. \end{aligned}$$

The relation Eq. (6.57) can be written in other forms, e.g. there is a version of formula Eq. (6.32) for this CY. If we combine Eq. (6.57) with the properties of $|{\mathcal {P}}_{(m_1,m_2)}|$

6.59 $$\begin{aligned} u + \frac{\epsilon }{2} \sum _{i<j} (s_{ij} + |{\mathcal {P}}_{(m_{1,ij}, m_{2,ij})}|)= & {} \sum _i \frac{{\mathcal {F}}(t + g_i \psi \cdot m_i+\frac{\epsilon }{2} \psi \cdot \sigma _i)}{g^2_i - (\epsilon /2)^2} \nonumber \\&+\sum _i \left[ g_i \left( \sum _v |K_{i,v}^{reg}|-\sum _e f_{\lambda _{i,e}} \right) \right. \nonumber \\&\left. -\sum _{e} |\lambda _{i,e}| (t_e + g_i \psi \cdot m_i+\psi \cdot \sigma _i \frac{\epsilon }{2}) \right] \nonumber \\&- \frac{1}{24} c_2(X)\cdot (nt + 2 \frac{\epsilon }{2} \sum _{i<j} \psi \cdot m_{ij}), \end{aligned}$$

where we used the property

6.60 $$\begin{aligned} -\frac{1}{24} c_2(X) \cdot (t + \psi \cdot m) + \frac{1}{24} c_2(X) \cdot t = \frac{m_1+m_2}{6}, \end{aligned}$$

which can be deduced from Eq. (5.24).

Finally we can use the geometrical engineering dictionary for *SU*(3) theory by identifying the Kähler parameters $(t_1,t_2,t_3)$ with two scalars $(\varphi _1, \varphi _2)$ in *SU*(3) vector multiplet and the coupling constant *h* as

6.61 $$\begin{aligned} t_1= h + 3 \varphi _1, \quad t_2 = 2 \varphi _1 - \varphi _2, \quad t_3 = 2 \varphi _2 - \varphi _1. \end{aligned}$$

We then match *exactly* the 7d and 5d master formulas Eqs. (3.5) and (5.39) by identifying

6.62 $$\begin{aligned} \begin{aligned} h_{1,\ell }&= -\sum _{i=1}^\ell m_{1,i} + \frac{1}{2} \sum _{i=1}^n m_{1,i} = \frac{1}{2} \left( -m_{1,1}-\cdots -m_{1,\ell }+m_{1,\ell +1} + \cdots + m_{1,n} \right) ,\\ h_{2,\ell }&= -\sum _{i=1}^\ell m_{2,i} + \frac{1}{2} \sum _{i=1}^n m_{2,i} = \frac{1}{2} \left( -m_{2,1}-\cdots -m_{2,\ell }+m_{2,\ell +1} + \cdots + m_{2,n} \right) \end{aligned} \end{aligned}$$

and imposing the conditions $m_{1*} =m_{2*}= 0$, which imply $h_{1,0}=h_{1,n}=0$, $h_{2,0}=h_{2,n}=0$ and amount to going from *U*(*n*) to *SU*(*n*) in 7d. We conclude that the partition function for 7d *SU*(*n*) theory on the given CY is the same as the partition function for 5d $SU(3)_0$ theory on $A_{n-1}$ space (both theories are extended to $S^1$ in the appropriate fashion). Using Eq. (6.61) the classical part Eq. (6.52) becomes

6.63 $$\begin{aligned} {\mathcal {F}} = - h(\varphi _1^2 + \varphi _2^2 - \varphi _1 \varphi _2 ) - \frac{4}{3} \varphi _1^3 - \frac{4}{3} \varphi _2^3 + \frac{1}{2} \varphi _1^2 \varphi _2 + \frac{1}{2} \varphi _1 \varphi _2^2 \end{aligned}$$

If instead we use Eq. (6.49) then we have

6.64 $$\begin{aligned} {{\mathcal {F}}} = - h(\varphi _1^2 + \varphi _2^2 - \varphi _1 \varphi _2 ) - \frac{4}{3} \varphi _1^3 - \frac{4}{3} \varphi _2^3 + \frac{1}{2} \varphi _1^2 \varphi _2 + \frac{1}{2} \varphi _1 \varphi _2^2 + f(\varepsilon ) h^3, \end{aligned}$$

which may correspond to adding some non-dynamical (purely geometric) term on 5d side.

## Conclusions and Speculations

Our main achievement in this paper is the 7d master formula, which we derive resting on two claims. The first, independence on 7d Coulomb branch parameters, has a deep meaning, both mathematically (compactness) and physically (properties of an index of M-theory). The second is more technical in nature, and has to do with factorization properties of ${\mathcal {F}}$. A better (equivariant) understanding of ${\mathcal {F}}$ and its (shift) properties, which will be discussed elsewhere, allows one to prove it. These properties are due to the interplay of ${\mathcal {F}}$ with D4-branes wrapping compact cycles, which play a crucial role in our correspondence. For geometries that admit a geometric engineering, the 7d master formula nicely matches the 5d one, extending the geometric engineering paradigm from $A_0$ to $A_n$ geometries.

### $TN_n$ 5d instanton partition function

Let us finish with a few remarks about the instanton partition function on $TN_n$ space. We showed that *SU*(*n*) 7d theory on CY is equivalent to 5d theory (which is prescribed by a given CY) on $A_{n-1}$ space with the following identification

7.1 $$\begin{aligned} m_{1,\alpha }= & {} h_{0,\alpha }-h_{1,\alpha }, \ldots , m_{n-1,\alpha } = h_{(n-2),\alpha } - h_{(n-1),\alpha },\quad \text {and} \nonumber \\ m_{n,\alpha }= & {} h_{(n-1),\alpha } + h_{0,\alpha } \end{aligned}$$

with

7.2 $$\begin{aligned} \sum _{i=1}^n m_{i,\alpha } = m_{*,\alpha } = 2 h_{0, \alpha }, \end{aligned}$$

where the parameter $\alpha$ stands for Cartan for 5d theory and $i=1,\ldots ,n$. For the case of 7d *SU*(*n*) (5d on $A_{n-1}$) we assume $h_{0,\alpha }=0$ and in this case both $m_{i,\alpha }$ and $h_{i,\alpha }$ are integers. For *U*(*n*) 7d theory we drop the traceless condition for *m*’s and the resulting theory should correspond to 5d theory on $TN_n$. If we take 7d master formula Eq. (5.39) and combine it with the above dictionary, we get the following conjecture for 5d partition function on $TN_n$

7.3 $$\begin{aligned}&Z_{SU(N)}^{5d} (TN_n \times S^1; z, \vec {b}, q_4, q_5) \nonumber \\&\quad = \sum _{\vec {h}_0} e^{f(2\vec {h_0})} \sum _{(\vec {h}_1, \ldots , \vec {h}_{n-1})} \prod _{i=1}^n Z^{5d}_{SU(N)} ({{{\mathbb {C}}}}^2 \times S^1; z, \vec {b}^{(i)} , q^{(i)}_4, q^{(i)}_5), \end{aligned}$$

where the function *f* is the same function that appears in Eq. (5.39), $(q^{(i)}_4, q^{(i)}_5)$ are defined in Eq. (3.6) and $\vec {b}^{(i)}$ in Eq. (3.7). In the case $m_*\ne 0$ we cannot claim that $h_{i,\alpha }$ are integers (but their appropriate differences are integers). The function $f(m_*)=f(2h_0)$ is a cubic polynomial in $m_*$ ($h_0$) and it can be calculated explicitly. However, the concrete form of *f* depends on 7d classical action Eq. (4.36), e.g. adding the term $g^{-1} ch_1$ to Eq. (4.36) simplifies *f* a bit. At the present level of understanding, for a given 7d classical action we can calculate the polynomial *f* explicitly. However we do not understand what the 5d interpretation of this term is. It is natural to expect that *f* can be absorbed into classical 5d terms. To illustrate this, let us rewrite $A_{n-1}$ case in Eq. (3.8) for $TN_n$

7.4 $$\begin{aligned} \beta ^{-1} \log \Big (Z^{5d}_{\mathrm{cl}} (TN_{n}\times S^1)\Big )= & {} \sum _{i=1}^n \frac{\Big \langle \vec \varphi + \vec {h}_i \epsilon _4^{(i)} + \vec {h}_{i-1} \epsilon _5^{(i)}, \vec \varphi + \vec {h}_i \epsilon _4^{(i)} + \vec {h}_{i-1} \epsilon _5^{(i)} \Big \rangle }{ \epsilon _4^{(i)} \epsilon _5^{(i)} } \nonumber \\= & {} \frac{\langle \vec \varphi + (\epsilon _5-\epsilon _4) \vec {h}_0, \vec \varphi + (\epsilon _5-\epsilon _4) \vec {h}_0\rangle }{n \epsilon _4 \epsilon _5} \nonumber \\&- \sum _{i=1}^n \Big \langle \vec {m}_i - \frac{\vec {m}_*}{n}, \vec {m}_i - \frac{\vec {m}_*}{n} \Big \rangle , \end{aligned}$$

where $\langle ,\rangle$ stands for the Lie algebra pairing. This simple calculation is suggestive but at the moment we cannot claim that we can do the same for all terms in *f*. We expect the answer to take the form Eq. (7.3), but we need a better 5d insight to fix ambiguities associated to *f*.

### Further directions

It would be desirable to construct the full equivariant background in M-theory. This would allow to completely fix the form of 7d classical action and fully justify our constructions. This background contains $G_4$ flux, which technically implies certain shift symmetry properties for ${\mathcal {F}}$. The fully equivariant definition of ${\mathcal {F}}$ (and of the twisted M-theory $\Omega$-background) and its interplay with $H^2$ vs $H^2_c$ is something we plan to address in the future.

We could replace ${{\mathbb {C}}}^2/{{\mathbb {Z}}}_n$ with a more general $\Gamma _{\mathfrak {g}} \subseteq SU(2)$, which by the McKay correspondence is classified by ADE7.5although the DT counterpart of this has not been fully developed.^17^ Here ${{\mathbb {Z}}}_n$ is the *n*-th cyclic group, ${\mathbb {D}}_n$ is the *n*-th binary dihedral group, ${\mathbb {T}}$ is the binary tetrahedral group, ${\mathbb {O}}$ is the binary octahedral group, and ${\mathbb {I}}$ is the binary icosahedral group.

More intriguing examples of our relations occur if we consider a hybrid setup for which one of the two manifolds is compact and the other is non-compact. For instance consider the case $M_4 = S^4$. On one side we have the index of a 5d SCFT, on the other we have the index of the 7d gravitational theory on $S^1 \times M_6^\sharp$, where $\sharp$ denotes resolution. Perhaps even more interesting is the case $M_4 = K3$, where we could learn about the physics of M-theory on K3 from studying partition functions of 5d SCFTs.

## ALE Spaces of $A_{n-1}$ Type

We collect basic information about ALE spaces of type $A_{n-1}$. They are hyperKähler manifolds that are the deformation (resolution) of ${{\mathbb {C}}}^2/{{\mathbb {Z}}}_n$. We are interested in their toric geometry.

### $A_1$ space

We start with the simplest example, namely $A_1$ type. First consider the singular space ${{\mathbb {C}}}^2/{{{\mathbb {Z}}}}_2$ with ${{\mathbb {Z}}}_2$-action on ${{\mathbb {C}}}^2$ $(z_1, z_2) \rightarrow (-z_1, -z_2)$. We can define invariant coordinates

A.1 $$\begin{aligned} x = z_1^2,\quad y = z_2^2,\quad w=z_1 z_2 \end{aligned}$$

with the relation

A.2 $$\begin{aligned} xy - w^2=0, \end{aligned}$$

which defines the singular $A_1$ space as a condition in ${{\mathbb {C}}}^3$. Alternatively we can define this space as quotient of ${{\mathbb {C}}}^3= (z_1, z_2, z_3)$ by ${{\mathbb {C}}}_*$-action with charges $(1, -2, 1)$

A.3 $$\begin{aligned} (z_1, z_2, z_3 )\rightarrow ( \lambda z_1, \lambda ^{-2} z_2, \lambda z_3) \end{aligned}$$

and introduce invariant coordinates

A.4 $$\begin{aligned} x= z_1^2 z_2,\quad y=z_3^2 z_2,\quad w= z_1 z_2 z_3 \end{aligned}$$

subject to the same condition in ${{\mathbb {C}}}^3$

A.5 $$\begin{aligned} xy - w^2 =0. \end{aligned}$$

The way to resolve this space is to remove the point $z_1=z_3=0$ and thus the resulting space is $O(-2) \rightarrow {\mathbb {C}}{\mathbb {P}}^1$, which is the same as $T^*{\mathbb {C}}{\mathbb {P}}^1$. This space is equipped with the well-known Eguchi–Hanson metric (hyperKähler metric) and it can be obtained either as Kähler reduction of ${{\mathbb {C}}}^3$ or as hyperKähler reduction of ${{\mathbb {C}}}^4$.

Since we are interested in the toric geometry of this space let us concentrate on the Kähler quotient picture. We can obtain this space by the Kähler quotient of ${{\mathbb {C}}}^3$ with respect to *U*(1) acting with charges $(1,-2,1)$. The corresponding moment map is

A.6 $$\begin{aligned} |z_1|^2 -2 |z_2|^2 + |z_3|^2 = t \end{aligned}$$

with $t>0$, which is related to the size of ${\mathbb {C}}{\mathbb {P}}^1$. The case $t=0$ corresponds to the singular space. The resulting space $A_1$ can be covered by two patches with coordinates

A.7 $$\begin{aligned} \begin{aligned} 1&\quad \Big (\xi _1^{(1)} = z_1^2 z_2 ,~ \xi _2^{(1)}=\frac{z_3}{z_1} \Big ), \\ 2&\quad \Big ( \xi _1^{(2)} =\frac{z_1}{z_3}, ~\xi _2^{(2)}= z_3^2 z_2 \Big ), \end{aligned} \end{aligned}$$

where on patch 1 we assume $z_1 \ne 0$ and on patch 2 $z_2 \ne 0$. On the intersection of two patches we have the coordinate change

A.8 $$\begin{aligned} \xi _1^{(2)} = \frac{1}{\xi _2^{(1)}}, \quad \xi _2^{(2)} = (\xi _1^{(2)})^{-2} \xi _1^{(1)}, \end{aligned}$$

which confirms that we deal with $O(-2)$ bundle over ${\mathbb {C}}{\mathbb {P}}^1$. There is a $T^2$-action on $A_1$ with two fixed points: patch 1 contains $(\xi _1^{(1)}, \xi _2^{(1)})=(0,0)$ (in ${{\mathbb {C}}}^3$-coordinates $z_3=0, z_2=0$) and patch 2 contains $(\xi _1^{(2)}, \xi _2^{(2)})=(0,0)$ (in ${{\mathbb {C}}}^3$-coordinates $z_1=0, z_2 =0$). If we define a $\mathbb {T}^3$-action on ${{\mathbb {C}}}^3$ as $z_i \rightarrow e^{\mathrm i\varepsilon _i} z_i$, we can read off the $T^2$ action on the homogeneous coordinates

A.9 $$\begin{aligned} \begin{aligned} 1&\quad \xi _1^{(1)} \rightarrow e^{i(2\varepsilon _1 + \varepsilon _2)} \xi _1^{(1)} ,\quad \xi _2^{(1)} \rightarrow e^{i(\varepsilon _3 - \varepsilon _1)} \xi _2^{(2)} , \\ 2&\quad \xi _1^{(2)} \rightarrow e^{i(\varepsilon _1 - \varepsilon _3)} \xi _1^{(2)},\quad \xi _2^{(2)} \rightarrow e^{i(2\varepsilon _3 + \varepsilon _2)} \xi _2^{(2)}, \end{aligned} \end{aligned}$$

and since we deal only with $T^2$-action it is convenient to define two independent $(\epsilon _4, \epsilon _5)$ such that $2 \epsilon _4= 2\varepsilon _1 +\varepsilon _2$ and $2\epsilon _5 = 2 \varepsilon _3 + \varepsilon _2$ or alternatively we can set $\varepsilon _2=0$ with the identification $\epsilon _4= \varepsilon _1$ and $\epsilon _5 = \varepsilon _3$. Therefore the fixed point data for the two fixed points is given by

A.10 $$\begin{aligned} (2\epsilon _4, \epsilon _5 - \epsilon _4),\quad (\epsilon _4 - \epsilon _5, 2\epsilon _5). \end{aligned}$$

If on ambient space ${{\mathbb {C}}}^3$ we define the standard Hamiltonian $H = \varepsilon _1 |z_1|^2 +\varepsilon _2 |z_2|^2 + \varepsilon _3|z_3|^2$ then we can evaluate its values at the fixed points of the quotient space. Using the DH theorem we can evaluate the equivariant volume of the quotient space as follows

A.11 $$\begin{aligned} \mathrm{vol} (A_1)= & {} \frac{e^{H_1}}{(\epsilon _5 - \epsilon _4) 2 \epsilon _4} + \frac{e^{H_2}}{(\epsilon _4 - \epsilon _5) 2 \epsilon _5} = \frac{1}{2\epsilon _4 \epsilon _5} - \frac{1}{4} t^2 \nonumber \\&- \frac{1}{12} (\epsilon _4 + \epsilon _5) t^3 + O(\epsilon ^2), \end{aligned}$$

where $H_1=\epsilon _4 t$ and $H_2=\epsilon _5 t$ are the values of Hamiltonian at the fixed points.

### $A_{n-1}$ space

We consider ALE spaces of type $A_{n-1}$. The singular $A_{n-1}$ space corresponds to ${{\mathbb {C}}}^2/{{\mathbb {Z}}}_{n}$ with ${{\mathbb {Z}}}_{n}$ generated by $\mathrm{diag} (e^{\frac{2\pi \mathrm i}{n}}, e^{-\frac{2 \pi \mathrm i}{n}})$ acting on $(z_1, z_2)$. We can define invariant coordinates

A.12 $$\begin{aligned} x = z_1^{n},\quad y = z_2^{n},\quad w=z_1 z_2 \end{aligned}$$

and realize $A_{n-1}$ singular space in ${{\mathbb {C}}}^3$ as

A.13 $$\begin{aligned} xy - w^{n}=0. \end{aligned}$$

Alternatively, we can think of $A_{n-1}$ as ${{\mathbb {C}}}^{n-1}_*$-quotient of ${{\mathbb {C}}}^{n+1}$ with charges

A.14 $$\begin{aligned} Q= \begin{pmatrix} 1 &{} -2 &{} 1 &{} 0 &{} \cdots &{} 0 &{} 0 &{} 0 &{} 0 \\ 0 &{} 1 &{} -2 &{} 1 &{} \cdots &{} 0 &{} 0 &{} 0 &{} 0 \\ \cdots &{} \cdots &{} \cdots &{} \cdots &{} \cdots &{} \cdots &{} \cdots &{} \cdots &{} \cdots \\ 0 &{} 0 &{} 0 &{} 0 &{} \cdots &{} 1 &{} -2 &{} 1 &{} 0 \\ 0 &{} 0 &{} 0 &{} 0 &{} \cdots &{} 0 &{} 1 &{} -2 &{} 1 \end{pmatrix}. \end{aligned}$$

Except for the first and last columns, this is (minus) the Cartan matrix of $A_{n-1}$. Assuming for ${{\mathbb {C}}}^{n+1}$ coordinates $(z_1, z_2, \ldots z_{n+1})$, we introduce invariant coordinates

A.15 $$\begin{aligned} \begin{aligned} x&= z_1^{n} z_2^{n-1} z_3^{n-2} \cdots z_{n+1}^0, \\ y&= z_1^0 z_2^1 z_3^2 \cdots z_{n+1}^{n},\\ w&= z_1 z_2 z_3 \cdots z_{n+1}, \end{aligned} \end{aligned}$$

which satisfy the relation in ${{\mathbb {C}}}^3$

A.16 $$\begin{aligned} xy-w^{n}=0. \end{aligned}$$

We are interested in the toric geometry of the resolved $A_{n-1}$ space, which can be realized as Kähler quotient ${{\mathbb {C}}}^{n+1}//U(1)^{n-1}$ with charge matrix Eq. (A.14) and moment maps

A.17 $$\begin{aligned} |z_\alpha |^2 - 2 |z_{\alpha +1}|^2 + |z_{\alpha +2}|^2 = t_{\alpha },\quad \alpha =1,2, \ldots , n-1, \end{aligned}$$

with $t_\alpha >0$ for the resolved $A_{n-1}$ space. The resolved $A_{n-1}$ space can be covered by *n* patches with the following homogeneous coordinates

A.18 $$\begin{aligned} \Big ( \xi _1^{(i)} = \frac{z_1^n z_2^{n-1} \cdots z_{n+1}^0}{(z_1 z_2 \cdots z_{n+1})^{i-1} },\quad \xi _2^{(i)} = \frac{z_1^0 z_2^1 \cdots z_{n+1}^n}{(z_1 z_2 \cdots z_{n+1})^{n-i}} \Big ) ,\quad i=1,2, \ldots , n, \end{aligned}$$

where all *z*’s are assumed to be non-zero except $z_{n+2-i}$ and $z_{n+1-i}$. At the intersection of patches *i* and $(i+1)$ we have the following coordinate change

A.19 $$\begin{aligned} \xi _1^{(i+1)} = \frac{1}{\xi _2^{(i)}},\quad \xi _2^{(i+1)} = (\xi _2^{(i)})^2 \xi _1^{(i)}, \end{aligned}$$

which should be compared to the case of $O(-2)$-bundle over ${\mathbb {C}}{\mathbb {P}}^1$, see Eq. (A.8). The space $A_{n-1}$ admits a $T^2$-action with *n* fixed points, with each patch *i* containing fixed point $(\xi _1^{(i)}, \xi _2^{(i)}) = (0,0)$ (or in ${{\mathbb {C}}}^{n+1}$ coordinates $z_{n+2-i}=0$ and $z_{n+1-i}=0$). Between fixed points on patch *i* and the nearby patch $(i+1)$ there is a ${\mathbb {C}}{\mathbb {P}}^1$ as can be seen from the coordinate transformations Eq. (A.19). Let us work out how $T^2$ acts around every fixed point by analyzing the quotient. Assuming $T^{n+1}$ action on ${{\mathbb {C}}}^{n+1}$ as $z_j \rightarrow e^{\mathrm i\varepsilon _j} z_j$ with $j=1,2,\ldots , n+1$ we can derive the toric action on the coordinates on patch *i*

A.20 $$\begin{aligned} \begin{aligned} \xi _1^{(i)}&\rightarrow e^{\mathrm i[ (n-i+1) \varepsilon _1 + (n-i) \varepsilon _2 + \cdots + (1-i) \varepsilon _{n+1}]} ~\xi _1^{(i)},\\ \xi _2^{(i)}&\rightarrow e^{\mathrm i[(i-n) \varepsilon _1 + (i-n+1) \varepsilon _2 + \cdots + i \varepsilon _{n+1}]} ~\xi _2^{(i)}. \end{aligned} \end{aligned}$$

Since the resulting symmetry is just $T^2$ we can choose two independent parameters for the global $T^2$. One can do the following choice $\varepsilon _2=\cdots =\varepsilon _n =0$ with the identification^18^

A.21 $$\begin{aligned} \epsilon _4 = \varepsilon _1,\quad \epsilon _5= \varepsilon _{n+1}. \end{aligned}$$

Using these global $\epsilon _4$ and $\epsilon _5$ we can derive the following local action

A.22 $$\begin{aligned} \epsilon _4^{(i)} = (n-i + 1) \epsilon _4 + (1-i) \epsilon _5,\quad \epsilon _5^{(i)} = (i-n) \epsilon _4 + i \epsilon _5, \end{aligned}$$

where we use notation $\xi _1^{(i)} \rightarrow e^{\mathrm i\epsilon _4^{(i)}} \xi _1^{(i)}$ and $\xi _2^{(i)} \rightarrow e^{\mathrm i\epsilon _5^{(i)}} \xi _2^{(i)}$ around fixed point $(\xi _1^{(i)}, \xi _2^{(i)}) = (0,0)$.

We are interested in the calculation of the equivariant volume using DH theorem

A.23 $$\begin{aligned} \mathrm{vol} (A_{n-1}) = \sum _{i=1}^n \frac{e^{H_i}}{\epsilon _4^{(i)} \epsilon _5^{(i)} } , \end{aligned}$$

where $H_i$ is the value of Hamiltonian at the fixed point *i* ($i=1,2,\ldots ,n$). If on ambient space ${{\mathbb {C}}}^{n+1}$ we define the Hamiltonian $H=\sum _{i=1}^{n+1} \varepsilon _i |z_i|^2$ then using above choices and the description of the fixed points we can derive the value of Hamiltonian at the fixed point *i* in terms of the values of the moment maps Eq. (A.17)

A.24 $$\begin{aligned} H_i= \epsilon _4 \sum _{j=1}^{n-i} j t_j + \epsilon _5 \sum _{j=1}^i (j-1) t_{n-j+1}, \end{aligned}$$

where we have $(n-1)$ *t*’s. Introduce $\alpha _i$ with $i=1,2,\ldots ,n$ such that

A.25 $$\begin{aligned} t_{n-i}=\alpha _{i+1} - \alpha _i, \end{aligned}$$

This map is not invertible unless we add an extra condition. It is natural to require

A.26 $$\begin{aligned} \sum _{i=1}^n\alpha _i=0. \end{aligned}$$

One can check that Eqs. (A.25) and (A.26) provide an invertible map between *t*’s and $\alpha$’s. Using Eq. (A.23) with the values of $H_i$ in Eq. (A.24) expressed in terms of $\alpha$’s, we get

A.27 $$\begin{aligned} \mathrm{vol} (A_{n-1})= & {} \frac{1}{n\epsilon _4 \epsilon _5} - \frac{1}{2} \sum _{i=1}^n\alpha _i^2 + \frac{\epsilon _4+\epsilon _5}{12} \sum _{i<j} (\alpha _i - \alpha _j)^3\nonumber \\&+ \frac{n(\epsilon _4-\epsilon _5)}{12} \sum _{i=1}^n \alpha _i^3 + O(\epsilon ^2). \end{aligned}$$

The first two terms on RHS were derived in [28]. The cubic term in $\alpha$’s has a nice form.

## $A_{n-1}$ versus $TN_n$

We collect information about the relation between the cyclic ALE spaces and ALF spaces.

The ALE space of type $A_{n-1}$ is the four-dimensional hyper-Kähler manifold obtained by the hyper-Kähler reduction of $\mathbb {H}^n \times \mathbb {H}$ with respect to $U(1)^n$ acting as

B.1 $$\begin{aligned} q_a \rightarrow q_a e^{i t_a},\quad w \rightarrow w e^{i \sum _{a=1}^n t_a}. \end{aligned}$$

The resulting metric is of the form

B.2 $$\begin{aligned} ds^2_{A_{n-1}} = \frac{1}{4} \tilde{V} d{\varvec{r}}^2 + \frac{1}{4} \tilde{V}^{-1} (d\tau +\chi )^2 \end{aligned}$$

where ${\varvec{r}} \in {{\mathbb {R}}}^3$, $\tau$ is periodic with period $4\pi$, and ${\varvec{x}}_a$ are the center’s positions in ${{\mathbb {R}}}^3$, such that ${\varvec{x}}_a \ne {\varvec{x}}_b$ ($a\ne b$) for non-singular space. We also use the following notations

B.3 $$\begin{aligned} \tilde{V} = \sum _{a=1}^{n} V_a, \quad V_a = \frac{1}{| {\varvec{x}}_a - {\varvec{r}} |} \end{aligned}$$

and

B.4 $$\begin{aligned} \chi = \sum _{a=1}^n \chi _a, \quad d\chi _a = \star _3 dV_a. \end{aligned}$$

For the case $n=1$ we recover the usual flat metric on ${{\mathbb {C}}}^2$ and thus we denote $A_0 = {{\mathbb {C}}}^2$. For the case $n=2$ the above metric is the well-known Eguchi–Hanson metric on $T^* {\mathbb {C}}{\mathbb {P}}^1$.

The cyclic ALF space, better known as multi-Taub-NUT space $TN_n$, is the four-dimensional hyper-Kähler manifold obtained by hyper-Kähler reduction of $\mathbb {H}^n \times \mathbb {H}$ wrt ${{\mathbb {R}}}^n$ acting as

B.5 $$\begin{aligned} q_a \rightarrow q_a e^{i t_a},\quad w \rightarrow w + R \sum _{a=1}^n t_a, \end{aligned}$$

with $R > 0$. The resulting metric has the form

B.6 $$\begin{aligned} ds^2_{TN_n} = \frac{1}{4} V d{\varvec{r}}^2 + \frac{1}{4} {V}^{-1} (d\tau +\chi )^2 \end{aligned}$$

with

B.7 $$\begin{aligned} V = \frac{1}{R^2} + \tilde{V}, \end{aligned}$$

where we use the same notations as before. Unlike $ds^2_{A_{n-1}}$ the metric on $TN_n$ is not asymptotically euclidean, instead at infinity it approaches ${{\mathbb {R}}}^3 \times S^1$ with *R* being the radius of the circle. In the limit $R \rightarrow \infty$ the metric $ds^2_{TN_n}$ goes to $ds^2_{A_{n-1}}$.

As hyper-Kähler manifolds $TN_n$ and $A_{n-1}$ are different, however as holomorphic symplectic manifolds (i.e., a complex manifold with symplectic (2, 0) form) they are the same [53]. Both metrics $ds^2_{TN_n}$ and $ds^2_{A_{n-1}}$ admit $T^2$-isometries with one particular *U*(1) being tri-holomorphic. We are interested in $T^2$ action on $A_{n-1}$ and $TN_n$. The detailed discussion of $T^2$ action on $A_{n-1}$ space has been presented in the previous appendix. Despite the fact that $TN_n$ is not toric (i.e., it cannot be glued from affine ${{\mathbb {C}}}^2$ patches) we believe that our discussion of $T^2$ action around fixed points (in particular Eq. (A.22)) goes through, since our previous analysis involves only complex coordinates and as complex manifolds these two spaces are the same. Intuitively this is clear since close to the origin (assuming that all centers ${\varvec{x}}_a$ are close to the origin), $TN_n$ is approximated by $A_{n-1}$.

Let us review some basic facts about the cohomologies of $TN_n$/$A_{n-1}$ and the line bundles over these spaces, following Witten [40]. The space $TN_n$ has two types of interesting cycles: compact 2-cycles $C_{a,b} \cong S^2$, which are fibered over the line segments joining the points with coordinates ${\varvec{x}}_a$ and ${\varvec{x}}_b$ in ${{\mathbb {R}}}^3$, and non-compact 2-cycles $C_a$ ($a=1,2,\ldots , n$). On $TN_n$ there are two versions of homology. The first version is topological $H_2 (TN_n, {{\mathbb {Z}}}) = {{\mathbb {Z}}}^{n-1}$, which is dual to the compactly supported cohomology $H^2_{\mathrm{cpct}} (TN_n, {{\mathbb {Z}}})$. Among all compact 2-cycles $C_{a,b}$ only $(n-1)$ are homologically independent and we can pick the standard basis

B.8 $$\begin{aligned} D_a = C_{a, a+1}, \quad a=1,2, \ldots , n-1 \end{aligned}$$

The intersection matrix is minus the Cartan matrix for $A_{n-1}$ group

B.9 $$\begin{aligned} ({{\mathcal {C}}})_{ab} = \begin{pmatrix} -2 &{} 1 &{} 0 &{} \cdots &{} 0 &{} 0 &{} 0 &{} 0 \\ 1 &{} -2 &{} 1 &{} \cdots &{} 0 &{} 0 &{} 0 &{} 0 \\ \cdots &{} \cdots &{} \cdots &{} \cdots &{} \cdots &{} \cdots &{} \cdots &{} \cdots &{} \\ 0 &{} 0 &{} 0 &{} 0 &{} \cdots &{} 1 &{} -2 &{} 1 \\ 0 &{} 0 &{} 0 &{} 0 &{} \cdots &{} 0 &{} 1 &{} -2 \end{pmatrix}. \end{aligned}$$

The second version is “geometrical” homology $H_2 (TN_n, {{\mathbb {Z}}}) = {{\mathbb {Z}}}^n$, which is generated by the non-compact cycles $C_a$ with intersection matrix $\langle C_a, C_b \rangle = \delta _{ab}$. We can define the following curvature two form $B_a=d \Lambda _a$, with

B.10 $$\begin{aligned} \Lambda _a = \frac{1}{2} \chi _a - \frac{V_a}{2V} (d\tau + \chi ), \end{aligned}$$

The $B_a$ are of (1, 1)-type (so anti-self dual) and

B.11 $$\begin{aligned} \frac{1}{2\pi } \int _{C_a} B_b = \delta _{ab}. \end{aligned}$$

Alternatively we have

B.12 $$\begin{aligned} \frac{1}{2\pi } \int _{C_{a,c}} B_b = \delta _{ab} - \delta _{bc}, \quad \frac{1}{2\pi } \int _{D_a} (B_b - B_{b+1}) ={{\mathcal {C}}}_{ab} . \end{aligned}$$

The curvature $B_a$ defines a line bundle ${{\mathcal {L}}}_a$ (correspondingly $m_a B_a$ defines ${{\mathcal {L}}}_a^{m_a}$). If we look at the sum $B=\sum _{a=1}^n B_a$ then *B* has vanishing integral over each compact cycle. However *B* is a normalizable harmonic two form and thus it is non-trivial in $L^2$-cohomology [54]. If we take the limit $R \rightarrow \infty$, then the form *B* is not normalizable on $A_{n-1}$ and there is no additional element in cohomology. Thus if we want to calculate the following integral

B.13 $$\begin{aligned} \int _{TN_n} c_1^2 ({{\mathcal {L}}}) = \sum _{a=1}^n m_a^2 \end{aligned}$$

where ${{\mathcal {L}}} = \oplus _{a=1}^m {{\mathcal {L}}}_a^{m_a}$ then the main difference between $TN_n$ and $A_{n-1}$ is the trace condition $\sum _a m_a =0$. On $A_{n-1}$ we have to impose the trace condition $\sum _a m_a =0$ since *B* is not normalizable and so it is not an element of $L^2$-cohomology.

## Useful Combinatorial Identities

In this appendix we collect the useful combinatorial identities that we use in the paper.

If we have two sequences of numbers $c_i$ and $d_i$ ($i=1,\ldots ,n$) the double sum can be reduced to a single sum as follows

C.1 $$\begin{aligned} \sum _{i<j} (c_i+c_j)(d_i-d_j)= & {} \sum _\ell c_\ell (d_1 +\cdots +d_{\ell -1} + d_\ell (n -2\ell +1) - d_{\ell +1}\nonumber \\&- \cdots -d_n). \end{aligned}$$

For the sequence of $m_i$ we define the short hand notation $m_{ij}=m_i - m_j$ and we have

C.2 $$\begin{aligned} \sum _{i<j} m_{ij} = \sum _{i=1}^n m_i (n+1-2i) = \sum _{i=1}^n \sigma _i (m), \end{aligned}$$

where $\sigma _i (m)$ is defined in Eq. (5.36). If we define $g_i$ as in Eq. (5.37) we have

C.3 $$\begin{aligned} \begin{aligned} \sum _{i=1}^n \frac{(g_i m_i+\frac{\epsilon }{2} \sigma _i)^2}{g_i^2 - (\epsilon /2)^2}&= \frac{n \epsilon ^2}{4g^2-(n\epsilon )^2} m_*^2 +\sum _{i=1}^n m_i^2 \\ \sum _{i=1}^n \frac{g_i m_i+\frac{\epsilon }{2} \sigma _i}{g_i^2 - (\epsilon /2)^2}&= \frac{g m_*}{g^2 - (n \epsilon /2)^2} \\ \sum _{i=1}^n \frac{1}{g_i^2 - (\epsilon /2)^2}&= \frac{n}{g^2 - (n \epsilon /2)^2} \end{aligned} \end{aligned}$$

and

C.4 $$\begin{aligned} \sum _{i=1}^n \frac{(g_i m_i+\frac{\epsilon }{2} \sigma _i)^3}{g_i^2 - (\epsilon /2)^2} = \frac{\epsilon }{2} \sum _{i<j} m_{ij}^3 +\frac{\epsilon ^2 g}{4g^2-(n\epsilon )^2} m_*^3 +g \sum _{i=1}^n m_i^3, \end{aligned}$$

where $m_*=\sum _i m_i$. If we have two sequences $m_{i,a}$ and $m_{i,b}$ (in our context the labels *a*, *b* are related to the faces of toric CY) then we have the following identities

C.5 $$\begin{aligned} \sum _{i=1}^n \frac{(g_i m_{i,a} +\frac{\epsilon }{2} \sigma _i(m_a)) (g_i m_{i,b} +\frac{\epsilon }{2} \sigma _i(m_b)) }{g_i^2 - (\epsilon /2)^2}= & {} \frac{n \epsilon ^2}{4g^2-(n\epsilon )^2} m_{*,a} m_{*,b}\nonumber \\&+\sum _{i=1}^n m_{i,a} m_{i,b} \end{aligned}$$

and

C.6 $$\begin{aligned}&\sum _{i=1}^n \frac{(g_i m_{i,a} +\frac{\epsilon }{2} \sigma _i(m_a))^2 (g_i m_{i,b} +\frac{\epsilon }{2} \sigma _i(m_b)) }{g_i^2 - (\epsilon /2)^2}\nonumber \\&\quad = \frac{\epsilon }{2} \sum _{i<j} m_{ij,a}^2 m_{ij,b} +\frac{\epsilon ^2 g}{4g^2-(n\epsilon )^2} m_{*,a}^2 m_{*,b} +g \sum _{i=1}^n m_{i,a}^2 m_{i,b} \end{aligned}$$

with $$m_{*,a}=\sum _i m_{i,a}$$ and $$m_{*,b}=\sum _i m_{i,b}$$.

## Footnotes

1 We work in K-equivariant setting, so the meaning of 7d is $S^1 \times X$. A similar remark applies to 5d.

2 Some progress can be made along the lines of Ref. [15].

3 Resolving the singularity gives rise to a flow to the Coulomb phase of the SCFT, which we denote $X_I$. The index *I* denotes possibly inequivalent resolutions of the singularity $M_6$ that correspond to different chambers in the Coulomb branch of the SCFT. The corresponding geometries are birational smooth CY3 related by flop transitions. Whenever $X_I$ admits a ruling supporting resolutions of ADE singularities, that phase of the CB geometry can be interpreted in terms of gauge theory. This is the case for the examples we consider in this paper, and for this reason we often omit the subscript *I* from $X_I$, as we are considering an explicit gauge theory phase as our $X$.

4 Or $(n-1)$ classes, there are some subtitles that we leave aside.

5 The Calabi–Yau condition is by no means necessary from the viewpoint of DT theory on threefolds, but it is useful when making contact with geometric engineering. What needs to be CY is the fivefold.

6 We often suppress powers of $\beta$, the radius of $S^1$, which can be restored by dimensional analysis.

7 Recall that, for a threefold *X*, $\dim H_4(X) = \dim H^2 (X,{{\mathbb {Z}}})_c$ by Poincaré duality, where we view compact support cohomology as $H^2(X,{{\mathbb {Z}}})_c \subset H^2_{dR}(X)_c$.

8 We temporarily switch to upper index *a* and lower index *i*, to match notations of that paper.

9 The $t=0$ limit, which features e.g. in Eq. (5.11), gives

10 Compared to Eq. (4.2), we introduce higher times $\tau _p ch_p$, which are discussed in Sect. 5.4, together with a proper treatment of $ch_0$. From $\sqrt{{\hat{A}}(X)}$ or ${\hat{\Gamma }}$-class, we only keep the term $-\frac{1}{24}c_2(X)\cdot t$.

11 Our assumption is actually a theorem for $X={{\mathbb {C}}}^3$ [49, 50].

12 Both here and when discussing the coupling of $ch_0$ we assume that *X* is CY, so that $q_{123}$ is constant. We believe the CY condition can be dropped, though details haven’t been worked out.

13 The full notation is $m_{i,f}$ where $i =1,\ldots ,n$ and $f \in \Delta _X^{(2)}$. Sometimes, we will drop indices.

14 This is a consequence of the fact that Eq. (4.14) is *a polynomial* in *q*’s [44], i.e. it cannot have singular terms as $\beta \rightarrow 0$.

15 This corresponds to setting $\varepsilon$ to zero for compact divisors. We do not need to make this specific choice and one can perform the analysis in general. However, the proper geometric treatment of this problem is beyond the scope of this work and will be explained elsewhere [44].

16 Reference [52] allows to extend the usual tools of Hodge theory to the non-compact CY3 setting.

17 In particular, it is unclear whether these will produce genuinely new invariants or not, which makes the question worth investigating.
